# Multi-Gene Phylogeny of the Ciliate Genus *Trachelostyla* (Ciliophora, Hypotrichia), With Integrative Description of Two Species, *Trachelostyla multinucleata* Spec. nov. and *T. pediculiformis* (Cohn, 1866)

**DOI:** 10.3389/fmicb.2021.775570

**Published:** 2022-02-01

**Authors:** Tengyue Zhang, Chen Shao, Tengteng Zhang, Weibo Song, Peter Vd’ačný, Saleh A. Al-Farraj, Yurui Wang

**Affiliations:** ^1^Laboratory of Protozoological Biodiversity and Evolution in Wetland, College of Life Sciences, Shaanxi Normal University, Xi’an, China; ^2^Institute of Evolution and Marine Biodiversity, Ocean University of China, Qingdao, China; ^3^Department of Zoology, Comenius University in Bratislava, Bratislava, Slovakia; ^4^Laboratory for Marine Biology and Biotechnology, Qingdao National Laboratory for Marine Science and Technology, Qingdao, China; ^5^Zoology Department, College of Science, King Saud University, Riyadh, Saudi Arabia

**Keywords:** ciliated protists, integrative taxonomy, ITS2 secondary structure, ontogenesis, phylogeny

## Abstract

Many hypotrich genera, including *Trachelostyla*, are taxonomically challenging and in a need of integrative revision. Using morphological data, molecular phylogenetic analyses, and internal transcribed spacer 2 (ITS2) secondary structures, we attempt to cast more light on species relationships within the genus *Trachelostyla*. The present multifaceted approach reveals that (1) a large-sized species with numerous macronuclear nodules, isolated from sandy littoral sediments in southern China, is new to science and is endowed here with a name, *T. multinucleata* spec. nov.; (2) two other Chinese populations previously identified as *T. pediculiformis* represent undescribed species; and (3) multigene phylogeny is more robust than single-gene trees, recovering the monophyly of the genus *Trachelostyla* with high bootstrap frequency. Additionally, ITS2 secondary structures and the presence of compensatory base changes in helices A and B indicate the presence of four distinct taxa within the molecularly studied members of the genus *Trachelostyla*. Molecular data are more suitable for delimitation of *Trachelostyla* species than morphological characters as interspecific pairwise genetic distances of small subunit (18S) rDNA, ITS1-5.8S-ITS2, and large subunit (28S) rDNA sequences do not overlap, whereas ranges of multiple morphometric features might transcend species boundaries.

## Introduction

Ciliates (phylum Ciliophora Doflein, 1901) are a large group of unicellular eukaryotes with various lifestyles, including endosymbiotic, epibiotic, and free-living (Van As and Basson, 2004; Bai et al., 2020; Wu et al., 2020; Foissner and Berger, 2021; Zhao et al., 2021). Hypotrichs (subclass Hypotrichia Stein, 1859) are not only one of the most morphologically differentiated ciliate groups, but also one of the most confused in terms of systematics and phylogeny (for reviews, see Berger, 1999, 2006, 2008, 2011; Chen L. et al., 2020, 2021; Paiva, 2020; Song et al., 2020; Xu W. et al., 2020; Xu Y. et al., 2020; Wang et al., 2020; Zhang et al., 2020a; Jung et al., 2021; Li et al., 2021a,b; Luo et al., 2021; Vd’ačný and Foissner, 2021). In the past decades, identification of hypotrichs mostly relied on interphase morphology, ontogenetic processes, and gene encoding for the small subunit (18S) rRNA molecule (Foissner, 2016; Song and Shao, 2017; Jung and Berger, 2019; Kaur et al., 2019; Kim and Min, 2019; Ma et al., 2021; Wang et al., 2021a). Recently, multigene analyses, including 18S rDNA, internal transcribed spacer (ITS) region (comprising ITS1, 5.8S, and ITS2), and large subunit (28S) rDNA provided more robust interpretations of phylogenetic relationships among hypotrichs than those based on a single gene (Dong et al., 2020; Lu et al., 2020). However, multigene data are available only for a minority of hypotrichs, and hence, increased marker sampling is needed to cast more light on the morphological evolution and systematics of hypotrichs.

*Trachelostyla* Borror, 1972, is one of many taxonomically “difficult” hypotrich genera. Distinguishing *Trachelostyla* species is difficult because the proposed taxa have a rather similar body size and shape as well as overlapping numbers of macronuclear nodules. This genus was introduced by Kahl (1932) and originally included two species, namely, *T. pediculiformis* (Cohn, 1866) and *T. caudata* Kahl, 1932. As none of them was fixed as type species, the erection of *Trachelostyla* was invalid at that time. Borror (1972) made the genus name available by fixing *T. pediculiformis* as the type species (Berger, 1999; Aescht, 2001). Due to the lack of type locality and information on some taxonomically important morphological features, Gong et al. (2006) neotypified *T. pediculiformis* and revised the diagnosis of *Trachelostyla*.

Currently, this genus is defined by a combination of the following features: a dorsoventrally flattened, nonspirally twisted, and elongated body with a peristomial region conspicuously narrowed; 11 cirri in the peristomial region (i.e., three frontal cirri, four frontoventral cirri, one buccal cirrus, and three postoral ventral cirri); one left and one right marginal cirral row not confluent posteriorly; and three caudal cirri. Gong et al. (2006) also established a phylogenetically related genus, *Spirotrachelostyla*, which differs from *Trachelostyla* by the spirally twisted body and about 13 cirri scattered in the anterior peristomial region. Three species (*Stichotricha simplex* Kahl, 1932; *Trachelostyla spiralis* Dragesco and Dragesco-Kernéis, 1986; and *Trachelostyla tani* Hu and Song, 2002) were originally included in *Spirotrachelostyla*.

*Trachelostyla* was reported by comparatively many researchers (for reviews, see Berger, 2008). However, the vast majority of reports did not provide detailed descriptions of the ciliary pattern based on silver-impregnated material. Some morphometric differences should not be, therefore, over-interpreted. In this light, Berger (2008) recognized only three valid species, namely, *T. caudata* (Kahl, 1932) Berger, 2008; *T. pediculiformis* (Cohn, 1866) Borror, 1972; and *T. rostrata* (Lepsi, 1962) Berger, 2008. Because of their overall morphological similarity as well as the lack of protargol-impregnated specimens and molecular data, some *Trachelostyla* populations were very likely misidentified, or their taxonomic status was at least questionable. For instance, as already mentioned by Berger (2008), a Chinese population of *T. pediculiformis* studied by Xu and Song (1999) conspicuously differs from the neotype population of Gong et al. (2006) in body size and the location of the rearmost postoral ventral cirrus, which is distinctly set off from the other two postoral cirri. Kahl’s (1928) population of *T. pediculiformis* was preliminarily classified as *incertae sedis* by Berger (2008) because it deviates from the typical *T. pediculiformis* by having two ventral cirral rows and two macronuclear nodules. On the other hand, *T. pediculiformis* does not exhibit any ventral cirral rows and possesses many scattered macronuclear nodules. Besides the interphase morphology, ontogenetic data might provide some clues for clearing taxonomic confusion as well. However, a detailed description of the whole ontogenetic process was reported only for a single *T. pediculiformis* population by Shao et al. (2007).

Previous phylogenetic studies suggest that 18S, ITS1-5.8S-ITS2, and 28S rDNA data potentially have the power to separate closely related hypotrich species (Huang et al., 2016; Li et al., 2017; Chen X. et al., 2021; Fan et al., 2021; Wang et al., 2021b). In the present study, we assessed the validity of phylogenetic relationships among *Trachelostyla* species, using a combination of morphological and multigene data as well as the secondary structure of the ITS2 molecule.

## Materials and Methods

### Sampling and Cultivation

*Trachelostyla multinucleata* spec. nov. was collected from the top 5 cm of sandy littoral sediments on the Xiaolajia Island in Daya Bay, near the city of Huizhou, southern China (22°36′40″N, 114°38′02″E) on April 1, 2018, when the water temperature was 26°C and the salinity was 33‰. *Trachelostyla pediculiformis* population 1 (pop. 1) was isolated from sediments and seawater collected from Tangdao Bay, Qingdao, China (35°55′57″N, 120°11′43″E) on November 5, 2017, when the water temperature was 15°C and the salinity was 32‰. Both samples were divided into aliquots that were used to establish raw cultures in Petri dishes at room temperature (23°C). Some rice grains were added to stimulate the growth of bacteria, which served as prey organisms for ciliates. Specimens from cultures were used for the subsequent molecular analyses.

### Taxonomic Methods and Terminology

*Trachelostyla multinucleata* spec. nov. and *T. pediculiformis* pop. 1 were investigated using a combination of detailed *in vivo* observation and protargol impregnation as described by Zhang et al. (2020b). Morphometric data were obtained from living and protargol-impregnated specimens. Illustrations of living cells were based on free-hand sketches and photographs. To distinguish parental and daughter structures during the morphogenetic processes, new (daughter) structures are painted solid, whereas old (parental) ciliary structures are depicted by contour. General terminology and classification follow Berger (2008).

Thirteen taxonomically important features were counted and measured on 21 protargol-impregnated specimens of *T. multinucleata* spec. nov., 11 individuals of *T. pediculiformis* pop. 1, and 16 cells of *T. pediculiformis* population 3 (pop. 3 from Huang et al., 2016; Table 1 and Supplementary Table 1). Morphometric data were processed in Python ver. 3.6.6 with the libraries NumPy (Oliphant, 2015) and Pandas (McKinney, 2010) to calculate pairwise similarities between all specimens using Gower’s coefficient. The pairwise Gower’s similarity matrix served as an input for the metric multidimensional scaling (MDS), which was conducted with the help of the SMACOF algorithm and the scikit-learn ver. 1.0 package^1^ (Pedregosa et al., 2011). MDS included 250 initializations each with 20,000 iterations. The MDS diagram was graphically prepared using the Matplotlib ver. 3.4.3 package (Hunter, 2007).

### DNA Extraction, PCR Amplification, and Sequencing

Extraction of the genomic DNA and PCR amplification of 18S, ITS1-5.8S-ITS2, and 28S rDNA as well as sequencing of *T. multinucleata* spec. nov. followed our previous study (Zhang et al., 2020b). Conspecific sequences obtained from multiple samples were identical, and therefore, only those derived from the single-cell sample were included in the subsequent phylogenetic analyses.

As for *T. pediculiformis* pop. 1, the single-cell genome isolation and amplification were carried out with the Repli-g single-cell kit (Qiagen, Hilden, Germany). PCR products were purified with AMPure XP beads (Beckman Coulter, IN, United States) and quantified by Qubit 3.0 Fluorometer (Invitrogen, Waltham, MA, United States). The final library was sequenced on the Illumina NovaSeq 6000 platform (Illumina, San Diego, CA, United States) with a 2 × 150 bp paired-end run in the Novogene Company (Beijing, China). Finally, rDNA sequences were extracted from assembled contigs. The quality of the rDNA contig was measured by Bowtie2 version 2.4.4 (Langmead and Salzberg, 2012) and Samtools version 1.14 (Danecek et al., 2021).

### Molecular Phylogenetic Methods

The newly obtained sequences were blasted against the nucleotide NCBI database^2^. The BLASTn algorithm revealed that they belong to the family Trachelostylidae (subclass Hypotrichia). All available trachelostylid sequences were carefully checked; some sequences designated as *Trachelostyla* sp. but without associated publications were excluded from the phylogenetic analyses and genetic distance analyses. Taxon sampling in the single-gene data set (18S rDNA) and multigene data set (18S + ITS1-5.8S-ITS2 + 28S rDNA) mostly followed Huang et al. (2016). Additionally, some other sequences published after the study of Huang et al. (2016) and related to *Trachelostyla* according to the BLASTn search were included in phylogenetic analyses as well. Four species (*Apodiophrys ovalis* Jiang and Song, 2010; *Diophrys scutum* (Dujardin, 1841) Kahl, 1932; *Paradiophrys zhangi* Jiang et al., 2011; and *Uronychia multicirrus* Song, 1997) were used as outgroup taxa, which follows Huang et al. (2016). GenBank accession numbers are shown in the respective figure and Supplementary Table 2. The sequences of *T. pediculiformis* populations 2 and 3 are from Huang et al. (2016). However, no 18S rDNA sequences are available from population 2, and thus it could not be included in the phylogenetic analyses.

Sequences were aligned online on the MAFFT ver. 7 server^3^ (Katoh et al., 2019), using the iterative refinement G-INS-i method, the gap opening penalty at 1.53, and the 200PAM/κ = 2 scoring matrix for nucleotide sequences. No masking strategy was used. The 5′ and 3′ ends of the resulting alignments were trimmed manually in the program BioEdit ver. 7.0 (Hall, 1999). SeaView ver. 4 was used to prepare the concatenated data set (Galtier et al., 1996; Gouy et al., 2010). The single-gene alignment contained 1,798 nucleotide positions, and the multigene data set comprises 3,678 positions. The reference alignments are provided in the Supplementary Material. The number of unmatched nucleotides and the pairwise *p*-distances of *Trachelostyla* and *Spirotrachelostyla* species were calculated with the help of the program BioEdit ver. 7.0 (Hall, 1999), using the sequence difference count matrix and sequence identity matrix options.

Maximum likelihood (ML) analyses were computed with the program IQ-TREE ver. 1.6.10 (Nguyen et al., 2015) on the IQ-TREE server^4^ (Trifinopoulos et al., 2016). Each molecular marker (i.e., 18S rDNA, ITS1-5.8S-ITS2 region, 28S rDNA) was assigned the best evolutionary substitution model as chosen by the in-built program under the Bayesian information criterion (Supplementary Table 3). Nodal support was assessed with 1,000 ultrafast bootstrap pseudo-replicates. All other parameters were left default. Bayesian inference was carried out in MrBayes ver. 3.2.7 (Ronquist et al., 2012) on the CIPRES portal ver. 3.3^5^ (Miller et al., 2010). Prior parameters of evolutionary models as estimated with IQ-TREE were implemented in Bayesian analyses with the “prset” command. Four Markov chain Monte Carlo simulations were run for 5,000,000 generations with a sampling frequency of 100 and a relative burn-in fraction of 25% (first 12,500 trees). Convergence of the MCMC analyses was confirmed in that the average standard deviation of split frequencies was well below 0.01, the potential scale reduction factor approached 1, effective sample sizes were >200, and no obvious trends were in the plots of generations vs. log probability. ML and BI trees were computed as unrooted and were rooted using the outgroup taxa in FigTree ver. 1.2.3^6^.

### Prediction of Internal Transcribed Spacer 2 Secondary Structure

Boundaries of ITS2 were determined according to Weisse et al. (2008) as well as Obert and Vd’ačný (2020). More specifically, we aligned trachelostylid sequences against *Meseres corlissi* (EU399522--29) and *Plagiotoma lumbrici* (MN176618--23), and then searched for the 5.8S-28S rRNA proximal stem of the ITS2 molecule. Predictions of the putative secondary structure of the ITS2 molecules included the formation of the imperfect 5.8S-28S rRNA helix, homology modeling, and free-energy minimization approach as implemented on the Mfold server ver. 3.0^7^ (Zuker, 2003). Thermodynamically optimal secondary ITS2 structures were manually processed in VARNA ver. 3.93 (Darty et al., 2009). The number of nucleotides in bulges and loops was counted and evaluated for each structural domain of the ITS2 molecules. Compensatory base changes (CBCs) were determined with the CBCAnalyzer option (Wolf et al., 2005) implemented in 4SALE ver. 1.7.1 (Seibel et al., 2006), and hemi-CBCs were searched for manually as recommended by Shazib et al. (2016). The tertiary structure of the ITS2 molecule was modeled using the online program RNAComposer ver. 1.0^8^ (Popenda et al., 2012).

## Results

### Zoobank Registration Numbers

Present work: urn:lsid:zoobank.org:pub:46848C5E-33DA-4C4B-B6CB-FD0B69F8FB88

*Trachelostyla multinucleata* spec. nov.:

urn:lsid:zoobank.org:act:9CFF6226-E9CF-4184-A7C3-67F2F5C00C64

### Systematics

Subclass Hypotrichia Stein, 1859
Family Trachelostylidae Small and Lynn, 1985
Genus *Trachelostyla* Borror, 1972

### *Trachelostyla multinucleata* Spec. nov.

#### Diagnosis

Size *in vivo* 200–310 × 35–60 μm. Body elongated, distinctly constricted in anterior fifth and slightly tail-like narrowed in posterior fifth. Macronuclear apparatus composed of about 60–90 nodules. Contractile vacuole located in posterior body fifth. Three frontal, one buccal, and four frontoventral cirri; three postoral ventral, two pretransverse, and five transverse cirri; three inconspicuous caudal cirri. One marginal cirral row on each side, composed of about 25–35 left and 30–45 right marginal cirri. Adoral zone slightly bipartite, composed of usually four apical ordinarily spaced membranelles and about 100 lapel narrowly spaced membranelles. Seven dorsal kineties with extremely long, spine-like dorsal cilia. Marine sandy sediment habitat.

#### Gene Sequences

The 18S rDNA, ITS1-5.8S-ITS2 region, and 28S rDNA sequences have been deposited in GenBank under the following accession nos.: MZ856308, MZ856304, and MZ856306, respectively.

#### Type Locality

Sandy littoral sediments from the Xiaolajia Island in the Daya Bay, near the city of Huizhou, southern China (22°36′40″N, 114°38′02″E).

**TABLE 1:** Morphometric data on *Trachelostyla multinucleata* spec. nov. (upper line), *T. pediculiformis* pop. 1 (middle line), and *T. pediculiformis* pop. 3 (lower line).

Character	H	Min	Max	Mean	Median	SD	CV	*n*
Body, length	290.0	210.0	340.0	291.8	290.0	32.6	11.2	20
	–	111.0	160.0	137.3	136.5	12.3	9.0	12
	–	86.0	161.9	124.1	125.5	18.1	14.5	16
Body, width	65.0	50.0	110.0	78.0	80.0	14.7	18.9	20
	–	26.0	66.0	46.1	47.0	11.0	24.0	12
	–	42.6	82.5	59.7	55.7	11.9	19.9	16
Body length:width, ratio	4.5	2.5	5.8	3.9	3.8	0.8	20.1	20
	–	2.0	4.3	3.1	3.0	0.7	23.1	12
	–	1.6	2.6	2.1	2.1	0.3	14.4	16
Buccal field, length	135.0	120.0	160.0	140.3	142.5	12.0	8.5	20
	–	55.0	78.0	67.9	67.5	5.7	8.4	12
	–	40.0	83.5	60.8	62.4	11.7	19.2	16
Buccal field, % of body length	46.6	40.3	57.1	48.4	48.2	4.0	8.2	20
	–	43.8	56.5	49.6	49.4	3.8	7.7	12
	–	40.1	59.9	48.9	48.5	5.9	12.0	16
Adoral membranelles, number	97	90	117	108.6	109.5	6.3	5.8	20
	–	42	46	43.8	43.5	1.3	3.1	12
	–	36	54	45.3	43.5	5.9	13.1	16
Buccal cirri, number	1	1	1	1.0	1.0	0	0	20
	–	1	1	1.0	1.0	0	0	20
	–	1	1	1.0	1.0	0	0	16
Frontal cirri, number	3	3	3	3.0	3.0	0	0	20
	–	3	3	3.0	3.0	0	0	20
	–	3	3	3.0	3.0	0	0	16
Frontoventral cirri, number	4	4	4	4.0	4.0	0	0	20
	–	4	4	4.0	3.0	0	0	20
	–	4	4	4.0	4.0	0	0	16
Postoral ventral cirri, number	3	3	3	3.0	3.0	0	0	20
	–	3	3	3.0	3.0	0	0	20
	–	3	3	3.0	3.0	0	0	16
Pretransverse ventral cirri, number	2	2	2	2.0	2.0	0	0	20
	–	2	2	2.0	3.0	0	0	20
	–	2	2	2.0	2.0	0	0	16
Transverse cirri, number	5	5	5	5.0	5.0	0	0	20
	–	5	5	5.0	3.0	0	0	20
	–	5	5	5.0	5.0	0	0	16
Left marginal cirri, number	29	25	35	30.3	30.0	2.7	8.8	20
	–	16	22	18.3	18.0	1.7	9.4	12
	–	17	26	20.0	19.0	2.7	13.4	15
Right marginal cirri, number	41	31	43	37.5	37.5	3.0	8.0	20
	–	23	30	25.9	25.5	2.2	8.3	12
	–	23	35	26.6	26.0	3.5	13.2	15
Caudal cirri, number	3	3	3	3.0	3.0	0	0	20
	–	3	3	3.0	3.0	0	0	20
	–	3	3	3.0	3.0	0	0	16
Macronuclear nodules, number	73	58	89	72.8	72.0	8.4	11.6	20
	–	7	16	13.3	14.5	3.5	26.6	12
	–	12	32	20.2	18.0	6.2	30.5	15
Macronuclei, largest diameter	5.9	3.8	7.9	5.3	5.3	1.0	18.1	20
	–	4.0	12.0	6.8	6.0	2.3	33.2	12
	–	4.6	10.8	7.4	7.0	1.7	23.2	16
Micronuclei, number	2	2	5	3.7	4.0	0.9	23.7	15
	–	2	2	2.0	2.0	0	0	11
	–	1	4	2.5	2.5	1.0	40.0	12
Micronuclei, largest diameter	3.4	2.6	4.2	3.2	3.2	0.4	17.7	15
	–	2.8	3.5	3.0	3.0	0.2	6.1	11
	–	1.7	2.8	2.3	2.3	0.4	15.6	12
Dorsal kineties, number	7	7	7	7.0	7.0	0	0	10
	–	6	6	6.0	6.0	0	0	6
	–	6	6	6.0	6.0	0	0	16

*All data based on protargol-impregnated specimens. Measurements in μm. H, holotype specimen; Min, minimum; Max, maximum; Mean, arithmetic mean; SD, standard deviation; CV, coefficient of variation in %; n, number of cells investigated.*

#### Type Material

A protargol slide (no. ZTY2018040106_1) with the holotype specimen marked with an ink circle and nine paratype slides (no. ZTY2018040106_2–10) as well as the DNA sample (ID Collection Code: C287) of a voucher specimen have been deposited in the Laboratory of Protozoology, Ocean University of China (OUC).

#### Etymology

The species-group name *multinucleata* is a composite of the stem of the Latin quantifier *mult*⋅*us* (many), the thematic vowel ⋅*i*-, and the Latin adjective *n
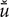
cle$\bar{a}$t*⋅*us*, *-a*, *-um* ([m; f; n], containing a nucleus), referring to the many macronuclear nodules whose number significantly exceeds that in other congeners.

### Morphological Description of *Trachelostyla multinucleata* Spec. nov.

**FIGURE 1:**
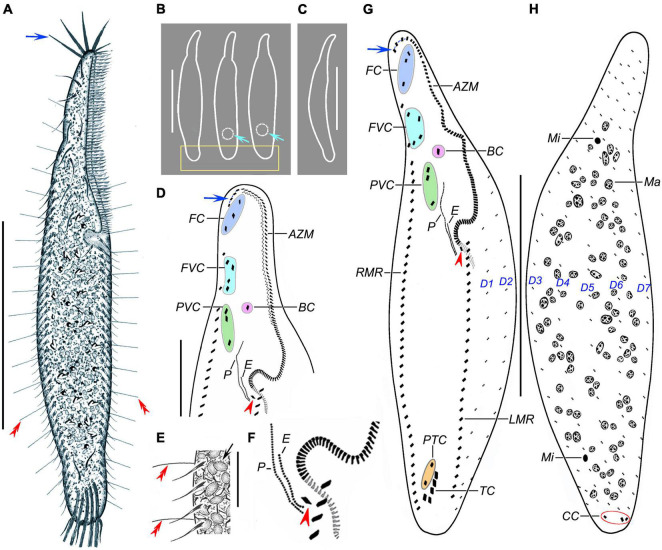
*Trachelostyla multinucleata* spec. nov. from life **(A–C,E)** and after protargol impregnation **(D,F,G,H)**. **(A)** Ventral view of a representative individual; blue arrow shows the four enlarged adoral membranelles, and red double-arrowheads mark dorsal bristles. **(B)** Overviews, showing different body shapes; light-blue arrows mark the contractile vacuoles, and the rectangle delimits the posterior body end that is tail-like in freshly collected specimens or broadly rounded in cultivated specimens. **(C)** Lateral overview. **(D)** Detail of the oral region; blue arrow shows four enlarged adoral membranelles, and the red arrowhead marks the first left marginal cirrus which is distinctly displaced rightward, hence appearing as an extra cirrus. **(E)** Ventral view, showing endoplasmic granules (black arrow), right marginal cirri, and dorsal bristles (red double-arrowheads). **(F)** Detail showing an extra cirrus (red arrowhead) right of the left marginal cirral row. **(G,H)** Ventral **(G)** and dorsal **(H)** views of holotype specimen, showing the ciliary pattern and the nuclear apparatus. Blue arrow shows the four enlarged adoral membranelles, red arrowhead marks the extra cirrus, and red ellipse (in **H**) delimits the three caudal cirri. AZM, adoral zone of membranelles; BC, buccal cirrus; CC, caudal cirri; E, endoral membrane; FC, frontal cirri; FVC, frontoventral cirri; LMR, left marginal cirral row; Ma, macronuclear nodules; Mi, micronuclei; P, paroral membrane; PTC, pretransverse ventral cirri; PVC, postoral ventral cirri; RMR, right marginal cirral row; TC, transverse cirri; D1–D7, dorsal kineties 1–7. Scale bars = 20 μm **(E)**, 50 μm **(D)**, and 130 μm **(A–C,G,H)**.

**FIGURE 2:**
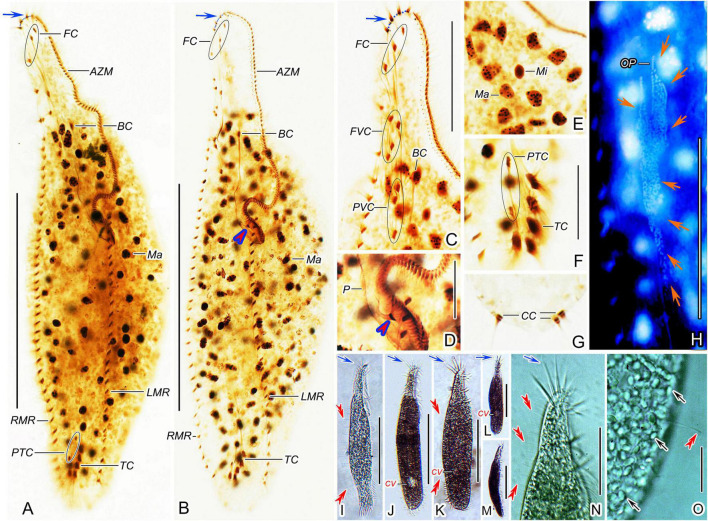
*Trachelostyla multinucleata* spec. nov. after protargol impregnation **(A–H)** and from life **(I–O)**. **(A,B)** Ventral views of holotype **(A)** and a paratype specimen **(B)**, showing the ciliary pattern and the nuclear apparatus. Blue arrows show the four enlarged adoral membranelles and red arrowhead indicates the extra cirrus above the buccal vertex. **(C,D)** Ventral views of the oral region. Blue arrow shows the four enlarged adoral membranelles and red arrowhead indicates the extra cirrus. **(E)** Macronuclear nodules and micronuclei. **(F,G)** Ventral **(F)** and dorsal **(G)** views of the posterior body region, showing prentransverse ventral and transverse cirri as well as caudal cirri. **(H)** Ventral view of an early divider, showing the opisthe’s oral primordium (red arrows). **(I–M)** Ventral **(I–L)** and lateral **(M)** overviews of different specimens, showing different body shapes, dorsal bristles (red double-arrowheads) and enlarged adoral membranelles (blue arrows). **(N)** Ventral view of the anterior body region, blue arrow shows the four enlarged adoral membranelles and red double-arrowheads mark dorsal bristles. **(O)** Dorsal view, showing endoplasmic granules (black arrows) and a dorsal bristle (red double-arrowhead). AZM, adoral zone of membranelles; BC, buccal cirrus; CC, caudal cirri; CV, contractile vacuole; FC, frontal cirri; FVC, frontoventral cirri; LMR, left marginal cirral row; Ma, macronuclear nodules; Mi, micronuclei; OP, opisthe’s oral primordium; P, paroral membrane; PTC, pretransverse ventral cirri; PVC, postoral ventral cirri; RMR, right marginal cirral row; TC, transverse cirri. Scale bars = 10 μm **(D–G)**, 20 μm **(O)**, 65 μm **(C,H,N)**, and 135 μm **(A,B,I–M)**.

Size of specimens from fresh raw cultures 205–310 × 35–60 μm, usually about 260 × 45 μm, length:width ratio ca. 5–6:1; body rather flexible but not contractile. Body shape elongated with narrowly rounded anterior end and broadly rounded posterior end; outline highly characteristic because tripartite: anterior body fifth distinctly constricted to a snout-like structure, trunk region cylindrical with more or less parallel margins, and posterior body fifth slightly tail-like narrowed (Figures 1A,B, 2I); posterior body constriction indistinct or missing in cultivated specimens, causing the body to appear bipartite (Figures 1B, 2J–L). Ventral side flat or slightly concave, dorsal side often arched in mid-body; dorsoventrally flatted 2:1 (Figures 1C, 2M); As many as 58–89 macronuclear nodules scattered throughout cytoplasm in trunk and tail-like region; individual nodules ovoid or ellipsoidal, 3.8–7.9 μm long after protargol impregnation, containing small chromatin bodies; two to five roughly globular micronuclei, 2.6–4.2 μm in largest diameter, scattered among macronuclei, sometimes difficult to recognize from smaller macronuclear nodules and exact number, thus, difficult to determine (Figures 1H, 2A,B,E and Table 1). Contractile vacuole located in posterior fifth of body length, approximately 18 μm across during diastole, usually very difficult to observe because cytoplasm studded with innumerable dark cytoplasmic inclusions and, thus, seen in about two out of the 13 specimens examined (Figures 1B, 2J–L). Cytoplasm colorless, packed with many granules 3–5 μm across, inclusions, and food vacuoles, rendering the cell with a dark and opaque appearance at low magnifications (Figures 2I–O). Cortex highly fragile, often does not withstand coverslip pressure, causing the cell to burst; no cortical granules recognizable. Crawls rapidly on debris particles, sometimes swims by rotation about main body axis.

Frontal-ventral-transverse cirral pattern and number of cirri constant, viz., cirri arranged in a 3:1:4:3:2:5 pattern (Figures 1D,G, 2A–C,F and Table 1). Cilia of frontal, buccal, frontoventral, postoral ventral, and pretransverse cirri 16–19 μm long; cilia of marginal cirri about 16 μm long; and cilia of transverse cirri 25–30 μm long. Three frontal cirri same in size as remaining cirri, obliquely arranged behind apical adoral membranelles. One buccal cirrus situated at level of mid-portion of adoral zone. Four frontoventral cirri located posterior to rearmost frontal cirrus and anterior to level of buccal cirrus, form a roughly rectangular pattern. Three postoral ventral cirri displaced distinctly anteriorly compared with most oxytrichids, i.e., located right anterior to paroral membrane, two anterior cirri conspicuously separated from third posterior cirrus (Figures 1D,G, 2C). Five transverse cirri distinctly enlarged and arranged in a J-shaped pattern caudally; two pretransverse cirri situated anterior to leftmost and rightmost transverse cirrus (Figures 1G, 2F). Right marginal cirral row commences slightly above anteriormost frontoventral cirrus and terminates slightly behind level of posteriormost transverse cirrus; two anteriormost cirri remarkably more widely spaced than remaining cirri (Figures 1D,G, 2A–C). Left marginal cirral row begins slightly above buccal vertex and terminates ahead of level of posteriormost transverse cirrus, marginal cirral rows, thus, not confluent posteriorly (Figures 1G, 2A,B); one extra cirrus (likely the first left marginal cirrus) situated at right of beginning of the left marginal cirral row and above the proximal end of the adoral zone of the membranelles, sometimes difficult to recognize in deeply impregnated specimens because they are easily hidden by deeply impregnated membranelles (Figures 1D,F,G, 2B,D, red arrowheads).

Dorsal bristles are very conspicuous because they are stiff and 15–18 μm long, arranged in seven longitudinal kineties (Figures 1A,E,H, 2I,K,N,O, red double-arrowheads). Number of dorsal bristles is determined only in two protargol-impregnated specimens: dorsal kinety 1 composed of 14 and 23 dikinetids, kinety 2 of 15 and 27 dikinetids, kinety 3 of 17 and 25 dikinetids, kinety 4 of 20 and 27 dikinetids, kinety 5 of 30 and 36 dikinetids, kinety 6 of 26 and 35 dikinetids, and kinety 7 of 32 and 45 dikinetids. Three caudal cirri, cilia of which are 11–14 μm long; middle and right cirrus usually close to each other and placed right of the cell’s midline, the left cirrus is situated left of the cell’s midline (Figures 1H, 2G).

Adoral zone of membranelles occupies about 40% of body length *in vivo* and approximately 48% in protargol preparations, divided into an apical and a lapel region. Apical region composed of four ordinarily spaced and radially extending membranelles, cilia 25–28 μm long (Figures 1A,D,G, 2A–C,I–L,N and Table 1). Lapel region, composed of 86–113 narrowly spaced membranelles, cilia up to 15 μm long, forms a *Gonostomum*-like pattern, i.e., runs along left body margin to level of mid-portion of paroral membrane, where it bends rather abruptly rightward, plunging into a comparatively long buccal cavity; a zigzag structure very likely composed of membranellar fibers recognizable right of lapel membranelles in some protargol-impregnated specimens (Figures 1D,G, 2A–D). Undulating membranes arranged almost in parallel, hence resembling a *Gonostomum*-like pattern. Paroral membrane commences left of postoral ventral cirri and runs to buccal vertex, distinctly longer than endoral membrane, about 35 μm long. Endoral membrane starts about in mid-portion of paroral membrane and terminates more or less at same level as paroral membrane, about 16 μm long (Figures 1D,F,G, 2D).

### Morphogenesis

**FIGURE 3:**
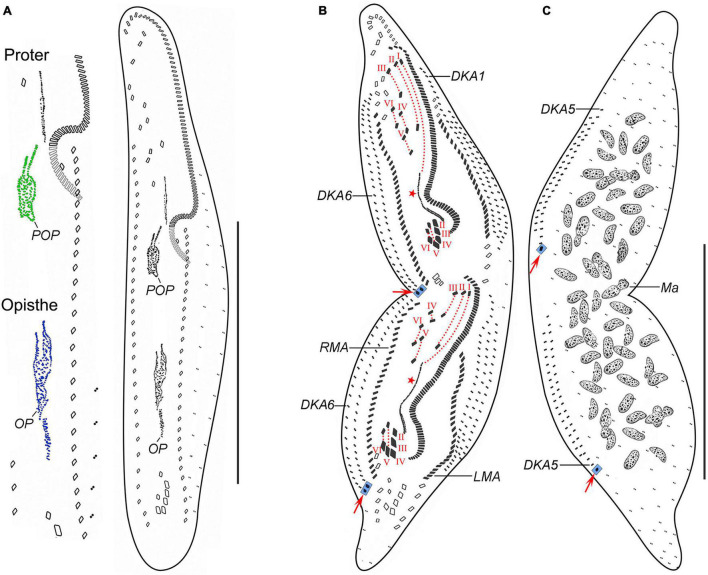
*Trachelostyla multinucleata* spec. nov., an early **(A)** and a late **(B,C)** divider after protargol impregnation. **(A)** Ventral view, showing the formation of oral primordia in the proter and the opisthe. **(B,C)** Ventral **(B)** and dorsal **(C)** views showing the cirri, dorsal kineties and cirral migration pattern as well as the nuclear apparatus. Red arrows mark caudal cirri, and red asterisks denote the undulating membranes anlagen. Frontal-ventral-transverse cirri originating from the same anlagen are connected by red dotted lines. DKA1, 5, 6, the first, fifth, and sixth dorsal kinety anlage; LMA, left marginal cirral anlage; Ma, macronuclear nodules; OP, opisthe’s oral primordium; POP, proter’s oral primordium; RMA, right marginal cirral anlage; I–VI, frontal-ventral-transverse cirri originating from anlagen I–VI. Scale bars = 150 μm **(A–C)**.

Only one early and one late divider were found in protargol preparations (Figures 2H, 3A–C). Morphogenesis commences apokinetally with the formation of two fields of densely arranged basal bodies, viz., the proter’s oral primordium situated near the buccal vertex and the opisthe’s oral primordium located below the mid-body (Figures 2H, 3A). All parental cirri appear intact at this stage and are, thus, apparently not involved in the formation of new oral primordia. In the late division stage, parental structures (i.e., adoral membranelles, undulating membranes, frontal-ventral-transverse cirri, marginal cirri, and caudal cirri) are almost resorbed and new structures are nearly completely developed both in the proter and the opisthe (Figures 3B,C).

Given the two dividers and multiple reorganizers, the following morphogenetic processes can be deduced for *T. multinucleata*: (**a**) the new 18 frontal-ventral-transverse (FVT) cirri are derived from six longitudinal anlagen (cirral streaks), generating 1, 3, 3, 3, 4, and 4 cirri, respectively, in both the proter and the opisthe (Figure 3B, red dotted lines); (**b**) the new frontal cirri are developed from the anteriormost cirri of the first three leftmost FVT cirral anlagen; (**c**) the undulating membranes are derived from the (leftmost) FVT cirral anlage I (Figure 3B, red asterisk); (**d**) the new buccal cirrus corresponds to the middle cirrus of the FVT cirral anlage II; (**e**) four frontoventral cirri originate from the middle cirrus of the FVT cirral anlage III, the anteriormost cirrus of anlage IV, and two anterior cirri of anlage VI; (**f**) three postal ventral cirri are derived from the middle cirrus of the FVT cirral anlage IV and two anterior cirri of anlage V; (**g**) two pretransverse ventral cirri are developed from the second posteriormost cirri of the FVT cirral anlagen V and VI; (**h**) each posteriormost cirrus of the FVT cirral anlagen II–VI migrates posteriorly to become a new transverse cirrus; (**i**) marginal cirral row anlagen develop within the parental cirral rows, and new structures completely replace the parental ones; (**j**) two dorsal kinety anlagen are formed right of the right marginal cirral anlagen, and five dorsal kinety anlagen are generated left of the left marginal cirral row; and (**k**) the right and the middle caudal cirrus originate from the posterior end of the two rightmost dorsal kinety anlagen, whereas the left caudal cirrus is derived from the rear end of the leftmost dorsal kinety anlage (Figures 3B,C, red arrows).

### Physiological Reorganization

**FIGURE 4:**
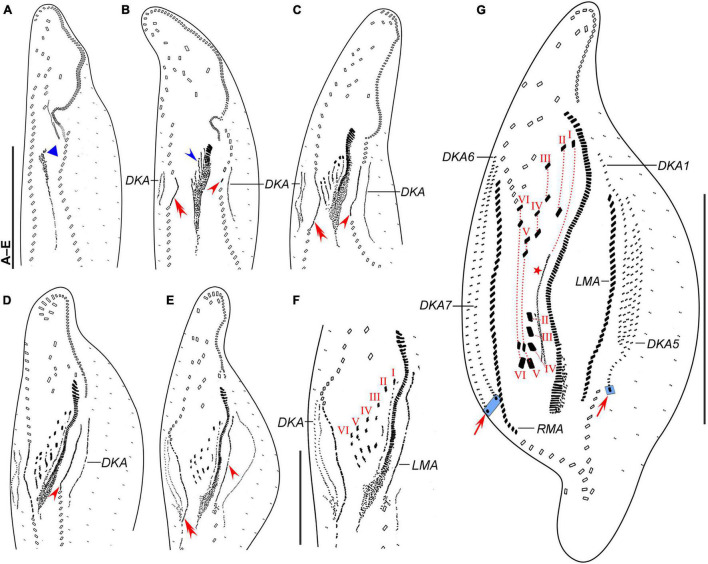
*Trachelostyla multinucleata* spec. nov., early **(A,B)**, middle **(C–F)**, and late **(G)** stages of physiological reorganization after protargol impregnation. **(A,B)** Ventral views to show the formation of the oral primordium (blue triangle), frontal-ventral-transverse cirral anlagen (blue arrowhead), right (red double-arrowhead) and left (red arrowhead) marginal cirral anlagen. **(C–E)** Ventral views, showing the formation of right (red double-arrowheads) and left (red arrowheads) marginal cirral anlagen and dorsal kineties anlagen. **(F)** Detail, showing the splitting pattern of frontal-ventral-transverse cirral anlagen I–VI. **(G)** Ventral view to show the formation of marginal cirral rows, caudal cirri (red arrows), dorsal kineties, and undulating membranes (red asterisk). Frontal-ventral-transverse cirri originating from the same anlagen are connected by red dotted lines numbered from left to right with I–VI. DKA1, 5, 6, 7, the first, fifth, sixth, and seventh dorsal kinety anlage; LMA, left marginal cirral anlage; RMA, right marginal cirral anlage; I–VI, frontal-ventral-transverse cirri originating from anlagen I–VI. Scale bars = 50 μm **(F)** and 120 μm **(A–E,G)**.

**FIGURE 5:**
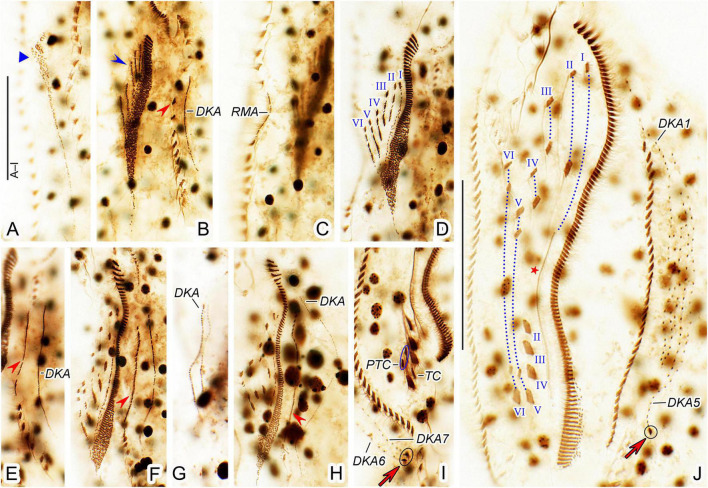
*Trachelostyla multinucleata* spec. nov., early **(A–C)**, middle **(D–H),** and late **(I,J)** stages of physiological reorganization after protargol impregnation. **(A–C)** Ventral views to show the formation of the oral primordium (blue triangle), frontal-ventral-transverse cirral anlagen (blue arrowhead), left (red arrowhead) and right marginal cirral anlagen. **(D–H)** Ventral views, showing the formation of left (red arrowheads) marginal cirral anlage and dorsal kineties anlagen. Frontal-ventral-transverse cirral anlagen are numbered from left to right with I–VI. **(I)** Detailed ventral view, red arrow marks two caudal cirri. **(J)** Ventral view of another late reorganizer to show the left caudal cirrus (red arrow), undulating membranes anlage (red asterisk), and dorsal kinety anlagen. Frontal-ventral-transverse cirri originating from the same anlagen are connected by red dotted lines and numbered from left to right with I–VI. DKA1, 5, 6, 7, the first, fifth, sixth, and seventh dorsal kinety anlage; PTC, pretransverse ventral cirri; RMA, right marginal cirral anlage; TC, transverse cirri; I–VI, frontal-ventral-transverse cirri originating from anlagen I–VI. Scale bars = 50 μm.

Physiological regeneration resembles divisional morphogenesis, but there is only one oral primordium and a single set of six FVT cirral anlagen. Two early stage (Figures 4A,B, 5A–C) and three middle-stage (Figures 4C–F, 5D–H) reorganizers as well as one late-stage reorganizer (Figures 4G, 5I,J) were found in protargol preparations.

The oral primordium is formed *de novo* as a longitudinal field of densely arranged basal bodies posterior to the buccal vertex (Figures 4A, 5A, blue triangles). Parental structures are not involved in the formation of new FVT cirral anlagen. These emerge as short streaks on the right side of the anterior half of the oral primordium (Figures 4B, 5B blue arrowheads). At this stage, also new adoral membranelles begin to organize at the anterior end of the oral primordium in a posteriad direction (Figures 4B, 5B). The new FVT cirral anlagen grow posteriorly along the differentiating adoral zone of membranelles to become longitudinal streaks. In middle-stage reorganizers, the six FVT streaks split new cirri in the following pattern 1, 3, 3, 3, 4, 4 (Figures 4C–G, 5D,F,H,J). The formation of 18 FVT cirri in reorganizers, thus, corresponds to the divisional morphogenesis shown in Figure 3B. The new undulating membranes are developed from the FVT cirral anlage I (Figures 4G, 5J, red asterisks).

Both new marginal cirral anlagen are generated within the mid-portion of the parental rows (Figures 4B–F, 5B,C,E,F,H, red arrowheads and red double-arrowheads). Subsequently, the new marginal cirral anlagen differentiate into marginal cirri in a posteriad direction to completely replace the parental ones (Figures 4G, 5J). The reorganization of the dorsal ciliature occurs in a unique way. Two parallel dorsal kinety anlagen are generated intrakinetally within the mid-portion of the parental dorsal kinety 7 (Figures 4B, 5G). They grow in a posteriad direction to become the new dorsal kineties 6 and 7 (Figures 4C–G, 5I). On the other hand, only a single anlage develops in the middle of the parental dorsal kinety 1 (Figures 4B–E, 5B,E,F,H). Later on, this anlage generates five dorsal kineties in the late reorganization stage (Figures 4G, 5J). Finally, a single caudal cirrus is differentiated at the posterior end of dorsal kinety 5 (Figures 4G, 5J, red arrows) and two caudal cirri are generated at the posterior end of dorsal kineties 6 and 7 (Figures 4G, 5I, red arrows).

### Morphological Redescription of the Qingdao Population of *Trachelostyla pediculiformis* (Cohn, 1866)

**FIGURE 6:**
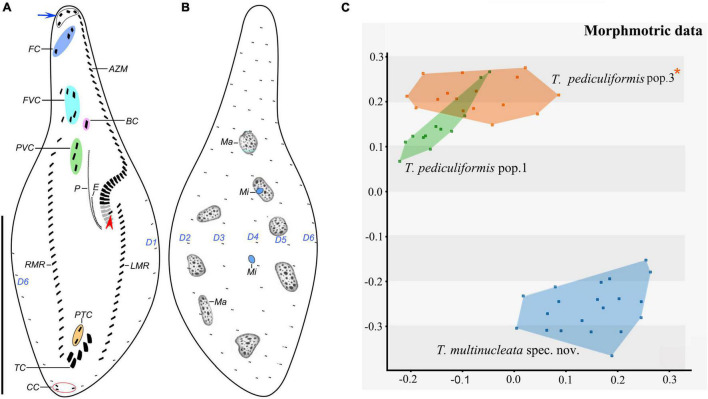
*Trachelostyla pediculiformis* pop. 1 after protargol impregnation **(A,B)** and multidimensional scaling of Gower’s similarity matrix based on morphometric data of three *Trachelostyla* species **(C)**. **(A,B)** Ventral **(A)** and dorsal **(B)** views of a representative specimen, showing the ciliary pattern and the nuclear apparatus. Blue arrow shows four enlarged adoral membranelles and red arrowhead marks the extra cirrus right of the left marginal cirral row. **(C)** MDS diagram of 48 individuals based on 13 morphometric features. *Trachelostyla pediculiformis* pop. 3 marked by red asterisk was a misidentified material in Huang et al. (2016). AZM, adoral zone of membranelles; BC, buccal cirrus; CC, caudal cirri; E, endoral membrane; FC, frontal cirri; FVC, frontoventral cirri; LMR, left marginal cirral row; Ma, macronuclear nodules; Mi, micronuclei; P, paroral membrane; PTC, pretransverse ventral cirri; PVC, postoral ventral cirri; RMR, right marginal cirral row; TC, transverse cirri; D1–6, dorsal kineties. Scale bar = 70 μm.

**FIGURE 7:**
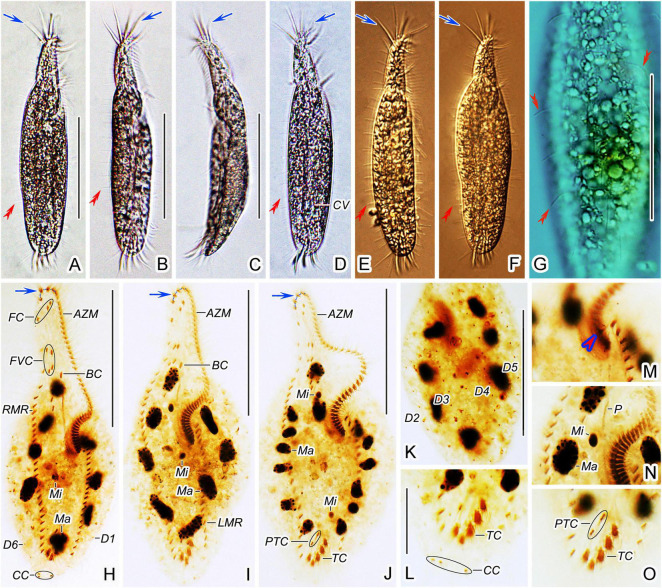
*Trachelostyla pediculiformis* pop. 1 from life **(A–G)** and after protargol impregnation **(H–O)**. **(A–F)** Ventral **(A,B,D–F)** and lateral **(C)** overviews of different individuals, showing the body shape and dorsal bristles (red double-arrowheads). Blue arrows show enlarged adoral membranelles. **(G)** Dorsal view, showing endoplasmic granules and dorsal bristle (red double-arrowheads). **(H–J)** Ventral views of different specimens, showing the ciliary pattern and the nuclear apparatus. Blue arrows show enlarged adoral membranelles. **(K)** Dorsal view. **(L,O)** Detailed ventral views of the posterior body region, showing the three caudal cirri, two pretransverse ventral cirri, and five transverse cirri. **(M,N)** Detailed ventral view of the proximal end of the adoral zone of membranelles to show the extra cirrus right of the left marginal cirral row (red arrowhead), the macronuclear nodules and micronuclei as well as the paroral membrane. AZM, adoral zone of membranelles; BC, buccal cirrus; CC, caudal cirri; CV, contractile vacuole; FC, frontal cirri; FVC, frontoventral cirri; LMR, left marginal cirral row; Ma, macronuclear nodules; Mi, micronuclei; P, paroral membrane; PTC, pretransverse ventral cirri; RMR, right marginal cirral row; TC, transverse cirri; D1–6, dorsal kineties 1–6. Scale bars = 25 μm **(L–O)** and 70 μm **(A–K)**.

Body size 105–150 × 20–40 μm *in vivo*, length:width ratio 4.0–5.5:1; body rather flexible but not contractile. Body elongated and bipartite, i.e., anterior body fifth distinctly snout-like constricted and trunk broadly cylindrical; anterior body end narrowly rounded while rear body end broadly rounded (Figures 6A,B, 7A,B,D–F). Ventral side flat, dorsal side slightly arched in mid-body; dorsoventrally flatted approximately 2:1 (Figure 7C). Seven to 16 ovoid or ellipsoidal macronuclear nodules, 3.5–12.0 × 3.0–8.7 μm in size after protargol impregnation, arranged in a ring-like pattern in trunk; two more or less globular micronuclei, 2.8–3.5 μm in diameter, usually one micronucleus located near anteriormost macronuclear nodules and one micronucleus near posteriormost nodules or in cell center (Figures 6B, 7H–J,N). Contractile vacuole located in posterior body fifth, approximately 15 μm across during diastole, difficult to recognize due to presence of numerous endoplasmic granules (Figure 7D). Cytoplasm colorless and packed with innumerable irregularly shaped granules 0.5–3.0 μm across, providing the cell with a grayish and opaque appearance at low magnification (Figures 7A–G). Cortex highly fragile; no cortical granules recognizable. Crawls rapidly on debris particles.

Frontal-ventral-transverse cirral pattern and number of cirri rather constant, i.e., three frontal cirri, four frontoventral cirri, one buccal cirrus, three postoral ventral cirri, two pretransverse cirri, and five transverse cirri (Figures 6A, 7H–J,L,O and Table 1). Cilia of all cirri are 6–9 μm long except for 13–17 μm-long transverse cirri. Right marginal cirral row begins at level of anteriormost frontoventral cirri; composed of 23–30 cirri; two anteriormost cirri conspicuously more widely spaced than the remaining ones. Left marginal cirral row commences slightly above buccal vertex; composed of 16–22 cirri including one extra cirrus (likely the first left marginal cirrus) right of beginning of left marginal cirral row; extra cirrus difficult to recognize in deeply impregnated specimens because situated above proximal end of adoral zone of membranelles (Figures 6A, 7M, red arrowheads). Marginal cirral rows are not confluent posteriorly, leaving a comparatively wide gap filled with enlarged transverse cirri (Figures 6A, 7H–J,L,O).

Dorsal bristles conspicuous because stiff and 7–8 μm long, arranged in six meridional kineties, dorsal kinety 1 distinctly shortened anteriorly while others bipolar (Figures 6A,B, 7A,B,D–G, red double-arrowheads and Table 1). Number of dorsal bristles determined in six protargol-impregnated specimens: dorsal kinety 1 composed of 9–11 dikinetids, kinety 2 of 10–12 dikinetids, kinety 3 of 12–14 dikinetids, kinety 4 of 13–16 dikinetids, kinety 5 of 16–18 dikinetids, and kinety 6 of 17–19 dikinetids (Figures 6A,B, 7H,K). Three caudal cirri, cilia about 6 μm long, middle and right cirrus typically close to each other and positioned right of cell’s midline, left cirrus situated left of cell’s midline (Figures 6A, 7H,L).

Adoral zone of membranelles occupies about 40% of body length *in vivo* and about 50% in protargol preparations; composed of 44 membranelles on average; divided into an apical and a lapel region (Figures 6A, 7A,B,D–F,H–J and Table 1). Apical region composed of three to five, 18–20 μm long, strong, and radially extending membranelles. Lapel membranelles narrowly spaced and extend along left margin of anterior body portion to plunge into a comparatively long buccal cavity, forming a *Gonostomum*-like pattern; length of membranellar cilia up to 10 μm (Figures 6A, 7A–F,H–J and Table 1). Undulating membranes arranged almost in parallel; paroral membrane begins at level of postoral anteriormost ventral cirri, extends to buccal vertex, and about 14 μm long in protargol preparations; endoral membrane commences about in mid-portion of paroral membrane, terminates at buccal vertex, and only about 6 μm long after protargol impregnation (Figures 6A, 7N).

### Morphometric Analyses

Morphological variation and boundaries of *Trachelostyla* species were analyzed using the multidimensional statistical approach and Gower’s similarity coefficient. MDS generated two clusters that were well separated along the second ordination axis. One cluster contained only specimens of *T. multinucleata* spec. nov., and the other cluster comprised individuals of *T. pediculiformis* pop. 1 and 3. Although there was a tendency to separate these two populations of *T. pediculiformis*, morphometric data were not sufficient to unambiguously delimit their boundaries (Figure 6C). This result, however, conflicts with the present phylogenetic, distance, and CBC analyses (see below) as both populations are genetically well separated and obviously represent distinct biological species.

### Phylogenetic Analyses

The rDNA sequences of *T. multinucleata* spec. nov. were obtained by the bidirectional Sanger sequencing, whereas the rDNA contig of *T. pediculiformis* pop. 1 (7,495 bp) was obtained from the assembled contigs of high-throughput sequencing. There were 176,857,976 raw reads in total, and 313,070 of them could be mapped to our rDNA contig with 95.3% similarity. The reads depth for this contig is 3,607, further proving its high quality and reliability. GenBank accession numbers and length of the nuclear 18S rDNA, ITS1-5.8S-ITS2, and 28S rDNA sequences of *T. multinucleata* spec. nov. and *T. pediculiformis* pop. 1 are summarized in Table 2.

**TABLE 2:** Origin, characterization, and GenBank accession numbers of trachelostylid species collected from Chinese coasts.

Species	Sampling location	SSU rDNA		ITS1-5.8S-IT2 region		LSU rDNA		References
					
		GenBank	Length	GenBank	Length	GenBank	Length	
*T. multinucleata* spec. nov.	Daya Bay, Huizhou, southern China (22°37′N, 114°38′E)	MZ856308	1592	MZ856304	455	MZ856306	1756	Present study
*T. pediculiformis* pop. 1	Tangdao Bay, Qingdao, northern China (35°57′N, 120°12′E)	MZ856309	1728	MZ856305	460	MZ856307	1826	Present study
*T. pediculiformis* neotype pop.	Bohai Sea, Tianjin, northern China (39°10′N, 117°10′E)	DQ057346	1769	KU594633	459	KU594633	1825	Gong et al., 2006, Huang et al., 2016
*T. pediculiformis* pop. 2	Mangrove Area, Shenzhen, southern China (22°31′N, 114°01′E)	n.a	n.a	KM222038	512	KM222127	1864	Gao et al., 2016, Huang et al., 2016
*T. pediculiformis* pop. 3	Jiaozhou Bay, Qingdao, northern China (36°06′N, 120°31′E)	KU594633	1723	KU594634	446	KU594653	1826	Huang et al., 2016
*Spirotrachelostyla tani*	Coast of Qingdao, northern China (36°08′N, 120°43′E)	FJ870093	1766	KU594632	457	KU594651	1829	Huang et al., 2016

*n.a., data not available.*

**FIGURE 8:**
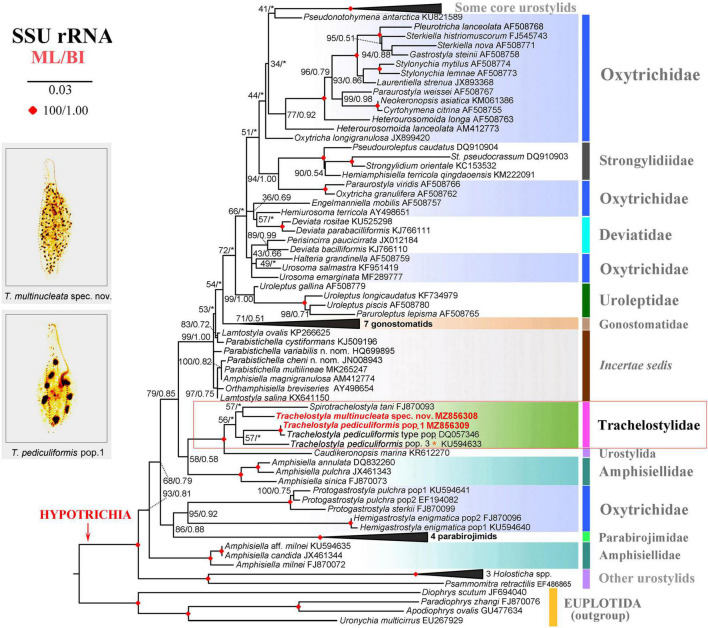
Maximum likelihood tree inferred from SSU rDNA sequences, showing the phylogenetic positions of *Trachelostyla multinucleata* spec. nov. and *Trachelostyla pediculiformis* pop. 1 (indicated by bold face and red color). Numbers near branches denote bootstrap values for maximum likelihood (ML) and posterior probabilities for Bayesian inference (BI). Black asterisks indicate the disagreement between ML and BI trees. GenBank accession numbers are provided after species names. *Trachelostyla pediculiformis* pop. 3 marked by red asterisk was very likely misidentified by Huang et al. (2016). Scale bar corresponds to three substitutions per 100 nucleotide positions.

**FIGURE 9:**
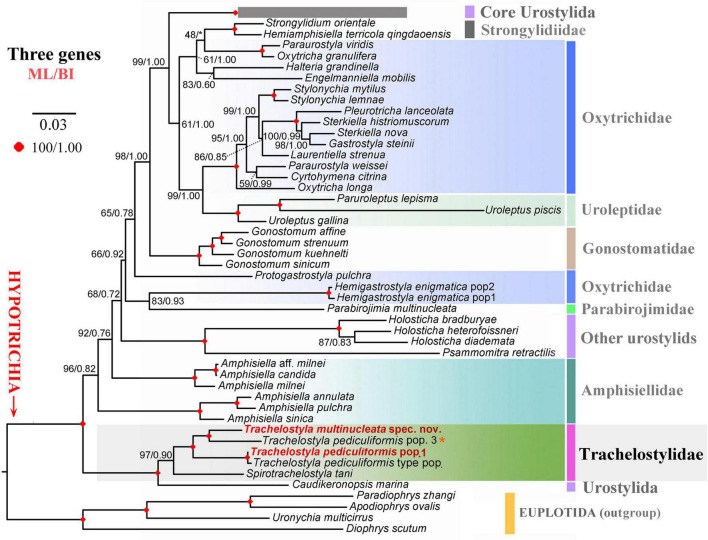
Maximum likelihood tree inferred from SSU rDNA, ITS region and LSU rDNA sequences, showing the phylogenetic positions of *Trachelostyla multinucleata* spec. nov. and *Trachelostyla pediculiformis* pop. 1 (indicated by bold face and red color). Numbers near branches denote bootstrap values for maximum likelihood (ML) and posterior probabilities for Bayesian inference (BI). Black asterisk indicates the disagreement between ML and BI trees. GenBank accession numbers are provided in Supplementary Table S2. *Trachelostyla pediculiformis* pop. 3 marked by red asterisk was a misidentified material in Huang et al. (2016). Scale bar corresponds to three substitutions per 100 nucleotide positions.

The phylogenetic positions of *T. multinucleata* spec. nov. and *T. pediculiformis* pop. 1 were determined with ML and BI. Topologies of ML and BI trees were nearly congruent, and hence, only the ML tree is shown with nodal supports from both methods (Figures 8, 9). Although the taxon sampling slightly differed between the single- and multigene data sets, *Trachelostyla* and *Spirotrachelostyla* consistently clustered with the urostylid *Caudikeronopsis marina* with full statistical support. The monophyletic origin of trachelostylids was strongly supported only in the multigene ML tree (97%), whereas it was very weakly supported in the single-gene ML tree (56%). Bayesian analyses provided only poor support (0.90) for monophyly of trachelostylids in the case of multigene data, whereas their common origin was not recognized in the case of single-gene data. The genus *Trachelostyla* was depicted as monophyletic in the multigene trees and paraphyletic in the single-gene trees. The reader is, therefore, referred to the fully resolved and strongly statistically supported multigene trees in which *Trachelostyla multinucleata* spec. nov. groups with *T. pediculiformis* pop. 3, and *T. pediculiformis* pop. 1 clusters with the neotype population of *T. pediculiformis* (Figure 9).

The number of unmatched nucleotide positions and the pairwise *p*-distances among members of the family Trachelostylidae are summarized in Table 3. The 18S rDNA sequence divergences among *T. multinucleata*, *T. pediculiformis* neotype pop., and *T. pediculiformis* pop. 1 and 3 ranged from 2.1% to 2.5%, corresponding to as many as 34–41 unmatched nucleotide positions. The observed genetic divergence in 18S rDNA sequences between *T. pediculiformis* pop. 1 and the neotype *T. pediculiformis* was 0.2% as there were only two unmatched nucleotide positions. The *p*-distances between *Trachelostyla* and *Spirotrachelostyla* species varied from 2.7% to 3.2%, i.e., there were 43–52 unmatched nucleotide positions (Table 4).

**TABLE 3:** Morphological comparison of some selected *Trachelostyla* species/populations.

Species	References	Body shape	Body length *in vivo* (μ m)	AZM	FVC	TC	Ma	LMC	RMC	DK
*T. multinucleata* spec. nov.	Present study	Elongate, with tail-like or round posterior end	205–310	90–117	11	5	58–89	25–35	31–43	7
*T. caudata*	Kahl, 1932	Elongate, with tail-like posterior end	150–220	n.a.	5	5	10	n.a.	n.a.	8–10
*T. caudata*	Kahl, 1933	Elongate, with tail-like posterior end	120–240	n.a.	5	5	>2	n.a.	n.a.	n.a.
*T. caudata*	Maeda and Carey, 1984	Elongate, with tail-like posterior end	156	n.a.	4	5	12?	n.a.	n.a.	n.a.
*T. caudata*	Carey, 1992	Elongate, with tail-like posterior end	150	n.a.	4	5	>2	n.a.	n.a.	n.a.
*T. caudata*	Al-Rasheid, 1999	Elongate, with round posterior end	150 (*in vivo* ?)	n.a.	n.a.	n.a.	>20	n.a.	n.a.	n.a.
*T. caudata*	Al-Rasheid, 2001	Elongate, with round posterior end	80–120	40	4	5	Many	n.a.	n.a.	n.a.
*T. pediculiformis* pop. 1	Present study	Elongate, with round posterior end	105–150	42–46	11	5	7–16	16–22	23–30	6
*T. pediculiformis*	Kahl, 1932	Elongate, with round posterior end	100–250	n.a.	8–11	5	>2	n.a.	n.a.	n.a.
*T. pediculiformis*	Biernacka, 1963	Elongate, with round posterior end	n.a.	n.a.	5	4	>2	n.a.	n.a.	n.a.
*T. pediculiformis* *	Borror, 1963	Elongate, with round posterior end	135	41	11 or 12	5	16–64?	23	32–35	3
*T. pediculiformis*	Kattar, 1970	Elongate, with round posterior end	150	n.a.	13	5	18	n.a.	n.a.	n.a.
*T. pediculiformis*	Jones, 1974	Elongate, with round posterior end	110	n.a.	13	5	26–30	n.a.	n.a.	n.a.
*T. pediculiformis*	Maeda and Carey, 1984	Elongate, with round posterior end	136–196	n.a.	10	5 or 6	>2	n.a.	n.a.	n.a.
*T. pediculiformis*	Carey, 1992	Elongate, with round posterior end	200	n.a.	10	5	>2	n.a.	n.a.	n.a.
*T. pediculiformis* neotype pop.	Gong et al., 2006	Elongate, with round posterior end	80–150	36–49	11	5	9–17	16–24	21–31	6

n.a., data not available. *Data from populations collected from sand, shell fragments, exoskeletons of isopods, dead oysters, and detritus were mixed together by Borror (1963). AZM, adoral zone of membranelles; DK, dorsal kinety; FVC, frontoventral cirri; Ma, macronuclei; LMC, left marginal cirri; RMC, right marginal cirri; TC, transverse cirri.

**TABLE 4:** Numbers of unmatched nucleotides (above diagonal) and pairwise *p*-distances (below diagonal) of SSU/ITS1-5.8S-ITS2/LSU rDNA sequences among members of the family Trachelostylidae.

Species	1.	2.	3.	4.	5.	6.
**1.** *T. multinucleata* spec. nov.		41/34/110	39/34/107	n.a/37/108	34/56/72	43/43/142
**2.** *T. pediculiformis* neotype pop.	0.025/0.075/0.063		2/0/9	n.a/18/52	40/68/110	50/40/162
**3.** *T. pediculiformis* pop. 1	0.024/0.075/0.061	0.002/0.000/0.006		n.a/18/49	38/68/107	48/40/159
**4.** *T. pediculiformis* pop. 2	n.a/0.081/0.062	n.a/0.040/0.030	n.a/0.040/0.028		n.a/61/98	n.a/37/167
**5.** *T. pediculiformis* pop. 3	0.021/0.124/0.041	0.025/0.149/0.063	0.024/0.149/0.061	n.a/0.133/0.056		52/69/139
**6.** *S. tani*	0.027/0.094/0.081	0.031/0.087/0.092	0.030/0.087/0.090	n.a/0.081/0.095	0.032/0.151/0.079	

*n.a., data not available.*

There was no difference in the ITS1-5.8S-ITS2 region between *T. pediculiformis* pop. 1 and the neotype *T. pediculiformis*. On the other hand, *p*-distances among *T. multinucleata*, *T. pediculiformis* neotype pop., *T. pediculiformis* pop. 1–3 varied from 4.0% to 14.9%, with 18–68 unmatched nucleotide positions. Distance between *Trachelostyla* and *Spirotrachelostyla* species ranged from 8.1% to 15.1% as there were 37–69 unmatched nucleotide positions (Table 4).

The *p*-distance in 28S rDNA sequences among *T. multinucleata*, *T. pediculiformis* neotype pop., *T. pediculiformis* pop. 1–3 spanned a range of 2.8%–6.3%, i.e., there were as many as 49–110 unmatched nucleotide positions. Genetic distances between *T. pediculiformis* pop. 1 and the neotype *T. pediculiformis* was 0.6%, which corresponds to nine unmatched nucleotide positions. The divergence between *Trachelostyla* and *Spirotrachelostyla* species varied from 7.9% to 9.5% with as many as 139–167 unmatched nucleotide positions (Table 4).

### Putative Internal Transcribed Spacer 2 Secondary Structure and Compensatory Base Change Analyses

**FIGURE 10:**
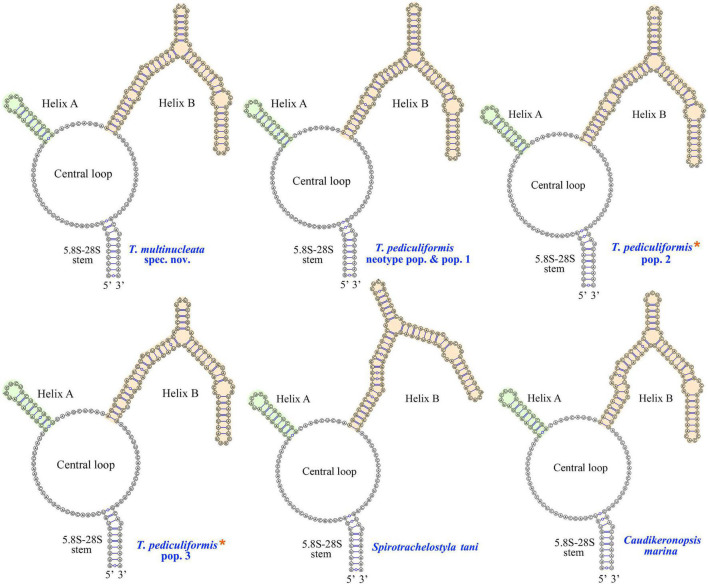
Putative secondary structures of the ITS2 molecule of five trachelostylids and *Caudikeronopsis marina*. *Trachelostyla pediculiformis* pop. 2 and 3 marked by red asterisks were two misidentified materials in Huang et al. (2016).

**FIGURE 11:**
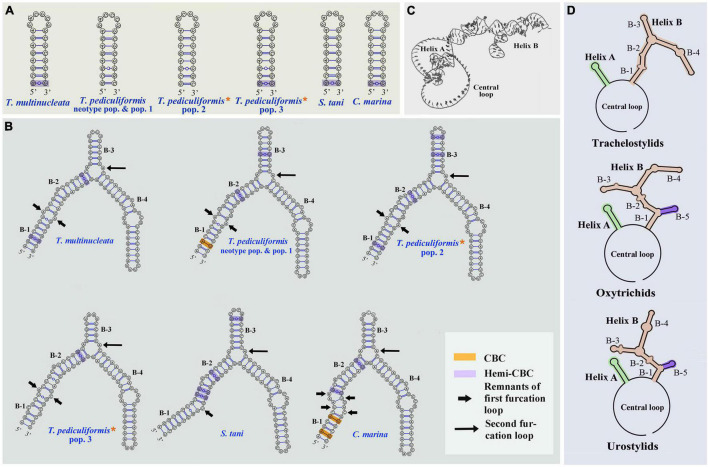
Putative models of ITS2 molecule. **(A,B)** Secondary structure of helix A and B of five trachelostylids and *Caudikeronopsis marina*. **(C)** Tertiary structure model proposed for *Trachelostyla multinucleata* spec. nov. **(D)** Schematic diagrams of the ITS2 secondary structure of trachelostylids, oxytrichids, and urostylids. *Trachelostyla pediculiformis* pop. 2 and 3 marked by red asterisks were very likely misidentified by Huang et al. (2016).

The ITS2 secondary structures of five *Trachelostyla* taxa and *Spirotrachelostyla tani* were proposed using Mfold and are shown in Figures 10, 11A,B. The tertiary structure model of the *T. multinucleata* ITS2 molecule was predicted based on the secondary structure model and is shown in Figure 11C. The length of ITS2 molecules ranges from 188 nt in *T. pediculiformis* pop. 3 to 194 nt in the *T. pediculiformis* neotype, *T. pediculiformis* pop. 1, and *T. pediculiformis* pop. 2. The trachelostylid ITS2 molecules consist of a central loop radiating two helices (A and B) of unequal length. The central loop is composed of 54 nucleotides in all taxa. Helix A is distinctly shorter than helix B and invariably consists of 22 nucleotides, forming eight pairs and a terminal hexaloop. Helix B displays a much more complex structure as it is composed of a furcation loop and four subhelices. Subhelix B-1 contains seven nucleotide pairs in all taxa except for *T. pediculiformis* pop. 3, which possesses six pairs instead due to one pyrimidine-pyrimidine mismatch. Subhelix B-2 is composed of one unpaired nucleotide (G) and 16 or 20 paired nucleotides. It is separated from subhelix B-1 by an adenine-adenine mismatch in all taxa except for *T. pediculiformis* pop. 3, which has an adenine-cytosine mismatch, and *S. tani*, which displays one unpaired uracil at the boundary of subhelices B-1 and B-2. Subhelix B-2 is separated from subhelices B-3 and B-4 by a furcation loop. Subhelix B-3 is the most variable among *Trachelostyla* and *Spirotrachelostyla* species in both length and nucleotide composition. This subhelix contains four nucleotide pairs in *T. pediculiformis* pop. 3, five pairs in *T. multinucleata*, six pairs in *S. tani*, and seven pairs in *T. pediculiformis* pop. 2 and *T. pediculiformis* (both the neotype and pop. 1). Subhelix B-4 is the longest with 16 nucleotide pairs, two bulges, and a terminal tetraloop.

The numbers of unmatched nucleotides and the counts of compensatory base changes between *Trachelostyla* and *Spirotrachelostyla* species are summarized in Supplementary Table 4. There was one CBC detected in helix B between *T. pediculiformis* (neotype pop. and pop. 1) and *T. pediculiformis* pop. 3 as well as between *T. pediculiformis* (neotype pop. and pop. 1) and *S. tani* (Supplementary Table 4). Hemi-CBCs were found in both helices and between all species (Figures 11A,B). The high number of unmatched nucleotides as well as the presence of CBCs and hemi-CBCs document that *T. pediculiformis* (neotype pop. and pop. 1), *T. pediculiformis* pop. 2, *T. pediculiformis* pop. 3, and *T. multinucleata* represent a distinct biological species each. As expected, no CBCs were found between the neotype population and population 1 of *T. pediculiformis* supporting their conspecificity.

## Discussion

### Establishment of *Trachelostyla multinucleata* Spec. nov.

Our new species undoubtedly belongs to the genus *Trachelostyla* as documented by the nonspirally twisted and elongated body with a conspicuously narrowed peristomial region, the 11:2:5:3 cirral pattern (i.e., 11 cirri in the frontal region, two pretransverse cirri, five transverse cirri, and three caudal cirri), and the *Gonostomum*-like oral apparatus. Berger (2008) based the taxonomy of the genus *Trachelostyla* on the morphology of the posterior body end and the structure of the nuclear apparatus. As mentioned, he recognized three valid species: *T. caudata*, *T. pediculiformis*, and *T. rostrata*.

Although *Trachelostyla caudata* has been reported several times, all descriptions are based only on live observations, and hence, no type material is available. As its specific epithet indicates, *T. caudata* has a tail-like posterior body end, which is also a property of *T. multinucleata*. Nevertheless, *T. multinucleata* can be distinguished from all populations of *T. caudata* by the much larger body (205–310 μm vs. 120–240 μm according to Kahl, 1932, 1933, 150–156 μm according to Maeda and Carey, 1984; Carey, 1992, and Al-Rasheid, 1999, and 80–120 μm according to Al-Rasheid, 2001) and the distinctly higher number of macronuclear nodules (58–89 vs. around 10 according to Kahl, 1932, 12 according to Maeda and Carey, 1984, more than 20 according to Al-Rasheid, 1999; Table 3).

*Trachelostyla multinucleata* can be separated from *T. pediculiformis* and *T. rostrata* by the much higher number of macronuclear nodules (58–89 nodules in *T. multinucleata* vs. 9–17 nodules in the *T. pediculiformis* neotype and two nodules in *T. rostrata*). According to Borror (1963), the number of macronuclear nodules in *T. pediculiformis* is unusually variable ranging from 16 to 64. This wide range needs to be, however, taken with caution because Borror (1963) very likely mixed multiple species that are not conspecific with *T. pediculiformis* and that even originated from different habitats including sand, shell fragments, exoskeletons of isopods, dead oysters, and detritus. Further comparisons with Borror (1963) populations are, unfortunately, not possible as their morphological descriptions are very insufficient. Finally, *T. multinucleata* has a tail-like narrowed posterior body end (fresh material is needed), and *T. pediculiformis* and *T. rostrata* display a broadly rounded rear body end. The morphometrical distinctness of *T. multinucleata* from *T. pediculiformis* is also shown in the MDS diagram (Figure 6C). Their separation is strongly corroborated also by genetic data in that the pairwise *p*-distances between *T. multinucleata* and the *T. pediculiformis* neotype are 2.5% in 18S rDNA, 7.5% in ITS region, and 6.3% in 28S rDNA sequences (Table 4).

### Species Identity of Previous *Trachelostyla pediculiformis* Population 2 and 3

Huang et al. (2016) assigned their populations 2 and 3 to *T. pediculiformis* due to their high morphological similarity with the *T. pediculiformis* neotype population. Indeed, the present multivariate analyses show that population 3 cannot be unambiguously differentiated from *T. pediculiformis* even when a combination of 13 morphometric characters is considered (Figure 6C and Supplementary Table 1). Unfortunately, detailed morphometric data are not available for population 2 and, therefore, its similarity with *T. pediculiformis* could not be statistically assessed. Despite that fact, populations 2 and 3 genetically differ so conspicuously from *T. pediculiformis* neotype pop. and *T. multinucleata* spec. nov. that they cannot be conspecific (Figures 8, 9 and Table 4).

*Trachelostyla pediculiformis* pop. 2 differs from *T. multinucleata*, *T. pediculiformis* neotype pop., and *T. pediculiformis* pop. 3 by 8.1% (37 unmatched nucleotides), 4.0% (18 unmatched nucleotides), and 13.3% (61 unmatched nucleotides) in ITS1-5.8S-ITS2 rDNA sequences, respectively, as well as by 6.2% (108 unmatched nucleotides), 3.0% (52 unmatched nucleotides), and 5.6% (98 unmatched nucleotides) in 28S rDNA sequences, respectively (Table 4).

*Trachelostyla pediculiformis* pop. 3 differs from *T. multinucleata* and *T. pediculiformis* neotype pop. by 2.1% (34 unmatched nucleotides) and 2.5% (40 unmatched nucleotides) in 18S rDNA sequences, 12.4% (56 unmatched nucleotides) and 14.9% (68 unmatched nucleotides) in ITS1-5.8S-ITS2 rDNA sequences, and 4.1% (72 unmatched nucleotides) and 6.3% (110 unmatched nucleotides) in 28S rDNA sequences (Table 4).

Although populations 2 and 3 are obviously not conspecific with the neotype population of *T. pediculiformis* and between each other, we await detailed morphological data to name them. Nevertheless, given the pronounced *p*-distances and the presence of CBC (Figures 10, 11A,B), they represent distinct biological species each.

### Morphological Versus Molecular Taxonomy of *Trachelostyla*

Multiple *Trachelostyla* populations were very likely misidentified in the past (for a review, see Berger, 2008). Even rather recently, two genetically distinct species, *Trachelostyla pediculiformis* pop. 2 and *T. pediculiformis* pop. 3, were misassigned to *T. pediculiformis* based on their high morphological similarity to the neotype population of *T. pediculiformis* (Gao et al., 2016; Huang et al., 2016). Indeed, the present multidimensional statistical approach could not unambiguously differentiate *T. pediculiformis* pop. 1 from *T. pediculiformis* pop. 3 even when a combination of 13 morphometric characters was employed. On the other hand, *T. multinucleata* spec. nov. could be morphometrically clearly separated from *T. pediculiformis* populations (Figure 6C). Despite this fact, multiple morphometric features transcend boundaries among *Trachelostyla* species (Tables 1, 3).

As molecular taxonomic methods are commonly available nowadays and molecular approaches are less complicated than morphological multivariate analyses, we propose a shift to molecular taxonomy and DNA barcoding to achieve more objective identification and delimitation of *Trachelostyla* species. Moreover, molecular data appear to be more suitable for delimitation of *Trachelostyla* species than morphological characters as interspecific pairwise genetic distances of 18S, ITS1-5.8S-ITS2, and 28S rDNA sequences do not overlap. On the other hand, multiple morphometric features might cross species boundaries (Tables 1, 3). Considering the *p*-distances between the *T. pediculiformis* neotype pop. and *T. pediculiformis* pop. 1, we suggest a 0.2% difference in 18S and a 0.6% divergence in 28S rDNA sequences as thresholds for discrimination of *Trachelostyla* species.

Also, CBC analysis of the ITS2 secondary structures might be of great help to more unambiguously delimit *Trachelostyla* species. The presence of CBCs strongly correlates with the existence of distinct biological species and, therefore, CBCs have been used as a reliable marker for species delimitation in various eukaryotes (e.g., Coleman, 2005, 2009; Wolf et al., 2005, 2013; Müller et al., 2007; Ruhl et al., 2010; Sorhannus et al., 2010). CBC-based species delimitation worked also well in multiple ciliate genera, for instance, in *Anoplophrya*, *Metaradiophrya*, *Paramecium*, *Pseudokeronopsis*, and *Vorticella* (Coleman, 2005; Sun et al., 2010, 2013; Zhan et al., 2019; Obert et al., 2021). On the other hand, no CBCs were detected among distinct *Spirostomum* (Shazib et al., 2016, 2019), *Trichodina* (Rataj and Vd’ačný, 2021), and clevelandellid (Pecina and Vd’ačný, 2021) species, which very likely corresponds to the shortness and high GC content of their ITS2 molecules. As concerns *Trachelostyla*, CBC and hemi-CBCs were observed among all *Trachelostyla* populations studied except the conspecific *T. pediculiformis* neotype pop. and pop. 1 (Figures 11A,B and Supplementary Table 4). This indicates that CBC analysis might be a powerful tool for delimitation of *Trachelostyla* species.

To summarize, the nuclear rDNA cistron (including 18S, ITS-5.8S-ITS2, and 28S rDNA sequences), the ITS2 secondary structure, and CBC analysis might be successfully utilized for identification and discrimination of *Trachelostyla* species as well as for uncovering new *Trachelostyla* species. Moreover, the combination of the multifaceted molecular approach might significantly reduce the risk of misidentification and subsequent errors in distributional and ecological analyses. On the other hand, some morphometric features might transcend species boundaries and even multivariate analyses might fail to separate genetically distinct taxa.

### Phylogeny of the Family Trachelostylidae

According to Berger (2008), the family Trachelostylidae comprises only two marine genera, viz., *Trachelostyla* and *Spirotrachelostyla*. Monophyly of trachelostylids was corroborated not only by morphological data but also by multigene phylogenetic analyses (Figure 9), albeit not by single-gene analyses (Figure 8). Various studies have already shown that concatenation of 18S, ITS1-5.8S-ITS2, and 28S rDNA sequences might lead to better resolved phylogenetic trees in the subclass Hypotrichia than 18S rDNA sequences *per se* (e.g., Huang et al., 2016; Obert and Vd’ačný, 2020; Zhang et al., 2020b; Wang et al., 2021b). The same applies also to the monophyletic origin of the genus *Trachelostyla*, which was only indicated in 18S rDNA phylogenies (Figure 8) although well recognizable in the multigene trees (Figure 9). Thus, when multiple molecular markers were employed, *Spirotrachelostyla* was robustly placed outside *Trachelostyla*. This is also consistent with morphological classifications as *Spirotrachelostyla* possesses a spirally twisted and spindle-shaped body, and *Trachelostyla* displays a nontwisted and bi- or tripartite body.

*Caudikeronopsis marina* was revealed to be a sister-group taxon of the family Trachelostylidae with full statistical support in both the single- and multigene phylogenies (Figures 8, 9). This result is surprising from a morphological viewpoint because *Caudikeronopsis* exhibits an urostylid ventral cirral pattern, and trachelostylids have an oxytrichid ventral pattern composed of more or less 18 FVT cirri.

Nevertheless, the mixing of taxa with urostylid and oxytrichid cirral patterns in molecular phylogenies is well known and was reconciled by the CEUU (Convergent Evolution of Urostylids and Uroleptids) hypothesis proposed by Foissner et al. (2004). Later on, Foissner and Stoeck (2006, 2008) and Kim et al. (2014) reported further taxa corroborating the CEUU hypothesis. The urostylid ventral cirral pattern very likely evolved or was lost multiple times independently during hypotrich evolution. The close kinship of *Caudikeronopsis* and trachelostylids is supported by the presence of caudal cirri, which are often lacking in the “core” urostylids. More importantly, *Caudikeronopsis* and trachelostylids share multiple unique deletions in the ITS2 molecule, which caused their helix B to be composed of only one furcation loop and four subhelices (Figures 10, 11A,B,D). By contrast, helix B contains two furcation loops and five subhelices in all other hypotrichs (Figure 11D) and oligotrichs studied so far (e.g., Coleman, 2005; Weisse et al., 2008; Li et al., 2013; Zhan et al., 2019; Obert and Vd’ačný, 2020). The single furcation loop of *Caudikeronopsis* and trachelostylids corresponds to the second furcation loop of oligotrichs and other hypotrichs (Figure 11D). The remnant of the first furcation loop is composed of an adenine-adenine mismatch in all trachelostylids except for *T. pediculiformis* pop. 3, which exhibits there an adenine-cytosine mismatch, and *S. tani*, which possesses only one unpaired uracil at the place of the former furcation loop (Figures 10, 11B). However, the vestige of the first loop is slightly larger and more complex in *Caudikeronopsis* (Figure 11B). The first furcation loop was apparently lost due to the complete deletion of subhelix B-5 already in the last common ancestor of *Caudikeronopsis* and trachelostylids. This loss also caused subhelices B-1 and B-2 to appear confluent in *Caudikeronopsis* and trachelostylids, whereas both subhelices are well recognizable in oligotrichs and all other hypotrichs (e.g., Coleman, 2005; Weisse et al., 2008; Li et al., 2013; Zhan et al., 2019; Obert and Vd’ačný, 2020).

## Data Availability Statement

The datasets presented in this study can be found in online repositories. The names of the repository/repositories and accession number(s) can be found in the article/Supplementary Material.

## Author Contributions

YW designed and supervised the research. TYZ and TTZ performed laboratory work. TYZ and PV analyzed data and wrote the manuscript. CS, WS, and SA-F revised the manuscript. All authors contributed to the article and approved the submitted version.

## Conflict of Interest

The authors declare that the research was conducted in the absence of any commercial or financial relationships that could be construed as a potential conflict of interest.

## Publisher’s Note

All claims expressed in this article are solely those of the authors and do not necessarily represent those of their affiliated organizations, or those of the publisher, the editors and the reviewers. Any product that may be evaluated in this article, or claim that may be made by its manufacturer, is not guaranteed or endorsed by the publisher.

## Footnotes

1
https://scikit-learn.org/stable/modules/generated/sklearn.manifold.MDS.html

2
https://www.ncbi.nlm.nih.gov/

3
http://mafft.cbrc.jp/alignment/server/

4
http://iqtree.cibiv.univie.ac.at/

5
http://www.phylo.org/

6
http://tree.bio.ed.ac.uk/software/figtree/

7
http://unafold.rna.albany.edu/?q=mfold/RNA-FoldingForm

8
http://rnacomposer.cs.put.poznan.pl/
