# Supplementary material for: Multi-Gene Phylogeny of the Ciliate Genus Trachelostyla (Ciliophora, Hypotrichia), With Integrative Description of Two Species, Trachelostyla multinucleata Spec. nov. and T. pediculiformis (Cohn, 1866)
Source: Front Microbiol. 2022 Feb 1;12:775570. doi: 10.3389/fmicb.2021.775570 (PMC8844511; doi:10.3389/fmicb.2021.775570)
Supplement: Supplementary file 1 [file Data_Sheet_1.docx]

**Electronic supplementary material**

**Multi-gene Phylogeny of the Marine Ciliate Genus *Trachelostyla* (Ciliophora, Hypotrichia), with Integrative Description of Two Species, *Trachelostyla multinucleata* spec. nov. and *T. pediculiformis* (Cohn, 1866)**

Tengyue Zhang^1,2,3^, Chen Shao^1^, Tengteng Zhang^2^, Weibo Song^1,2^, Peter Vďačný^3^, Saleh A. Al-Farraj^4^, Yurui Wang^1^*

^1^ Laboratory of Protozoological Biodiversity and Evolution in Wetland, College of Life Sciences, Shaanxi Normal University, Xi’an 710119, China

^2^ Institute of Evolution and Marine Biodiversity, Ocean University of China, Qingdao 266003, China

^3^ Department of Zoology, Comenius University in Bratislava, Bratislava 84215, Slovakia

^4^ Zoology Department, College of Science, King Saud University, Riyadh 11451, Saudi Arabia

* Correspondence wangyurui@snnu.edu.cn

______________________________________________________________________

Pages: 16

Tables: 4

Alignments: 3

**SUPPLEMENTARY TABLE S1 |** Morphometric data on 48 specimens belonging to three *Trachelostyla* species.

| **Species** | **Specimen** | **Body, length** | **Body, width** | **Body length/ width, ratio** | **Buccal field, length** | **Buccal field, % of body length** | **AM, number** | **Left marginal cirri, number** | **Right marginal cirri, number** | **Dorsal kineties, total number** | **MA nodules, number** | **MA, largest diameter** | **MI, number** | **MI, largest diameter** |
| --- | --- | --- | --- | --- | --- | --- | --- | --- | --- | --- | --- | --- | --- | --- |
| *T. multinucleata* | Tm1 | 290 | 65 | 4.5 | 135 | 46.6 | 111 | 30 | 35 | 7 | 73 | 5.9 | 2 | 3.4 |
| *T. multinucleata* | Tm2 | 270 | 80 | 3.4 | 130 | 48.1 | 102 | 29 | 41 | 7 | 76 | 5.2 | 4 | 2.6 |
| *T. multinucleata* | Tm3 | 310 | 75 | 4.1 | 145 | 46.8 | 113 | 28 | 31 | 7 | 58 | 5.0 | 2 | 2.8 |
| *T. multinucleata* | Tm4 | 280 | 85 | 3.3 | 140 | 50.0 | 98 | 30 | 37 | 7 | 68 | 6.1 | 5 | 3.0 |
| *T. multinucleata* | Tm5 | 290 | 65 | 4.5 | 155 | 53.4 | 105 | 25 | 37 | 7 | 75 | 5.6 | 4 | 3.2 |
| *T. multinucleata* | Tm6 | 300 | 80 | 3.8 | 145 | 48.3 | 110 | 34 | 43 | 7 | 81 | 5.8 | 4 | 4.2 |
| *T. multinucleata* | Tm7 | 325 | 80 | 4.1 | 160 | 49.2 | 112 | 32 | 38 | 7 | 72 | 6.3 | 3 | 3.1 |
| *T. multinucleata* | Tm8 | 270 | 60 | 4.5 | 120 | 44.4 | 109 | 27 | 33 | 7 | 62 | 7.9 | 4 | 3.2 |
| *T. multinucleata* | Tm9 | 260 | 60 | 4.3 | 145 | 55.8 | 117 | 32 | 40 | 7 | 71 | 5.9 | 4 | 3.0 |
| *T. multinucleata* | Tm10 | 335 | 95 | 3.5 | 150 | 44.8 | 115 | 33 | 35 | 7 | 82 | 6.5 | 4 | 2.9 |
| *T. multinucleata* | Tm11 | 320 | 65 | 4.9 | 145 | 45.3 | 108 | 32 | 39 | 7 | 64 | 5.4 | 4 | 2.8 |
| *T. multinucleata* | Tm12 | 290 | 75 | 3.9 | 145 | 50.0 | 110 | 28 | 37 | 7 | 86 | 4.6 | 5 | 3.3 |
| *T. multinucleata* | Tm13 | 305 | 90 | 3.4 | 145 | 47.5 | 107 | 28 | 37 | 7 | 65 | 4.0 | 4 | 3.2 |
| *T. multinucleata* | Tm14 | 295 | 90 | 3.3 | 130 | 44.1 | 116 | 35 | 43 | 7 | 70 | 5.0 | 3 | 3.4 |
| *T. multinucleata* | Tm15 | 290 | 50 | 5.8 | 140 | 48.3 | 90 | 29 | 35 | 7 | 70 | 3.8 | 4 | 3.2 |
| *T. multinucleata* | Tm16 | 340 | 110 | 3.1 | 160 | 47.1 | 106 | 30 | 37 | 7 | 63 | 5.0 | n.a. | n.a. |
| *T. multinucleata* | Tm17 | 335 | 80 | 4.2 | 135 | 40.3 | 109 | 35 | 38 | 7 | 84 | 4.7 | n.a. | n.a. |
| *T. multinucleata* | Tm18 | 210 | 70 | 3.0 | 120 | 57.1 | 113 | 31 | 39 | 7 | 75 | 5.3 | n.a. | n.a. |
| *T. multinucleata* | Tm19 | 240 | 95 | 2.5 | 120 | 50.0 | 111 | 29 | 36 | 7 | 89 | 4.0 | n.a. | n.a. |
| *T. multinucleata* | Tm20 | 280 | 90 | 3.1 | 140 | 50.0 | 109 | 29 | 38 | 7 | 72 | 4.9 | n.a. | n.a. |
| *T. pediculiformis* pop. 1 | Tp1-1 | 133 | 57 | 2.3 | 70 | 52.6 | 46 | 20 | 28 | 6 | 8 | 12.0 | 2 | 3.0 |
| *T. pediculiformis* pop. 1 | Tp1-2 | 140 | 52 | 2.7 | 75 | 53.6 | 43 | 18 | 26 | 6 | 16 | 5.0 | 2 | 3.0 |
| *T. pediculiformis* pop. 1 | Tp1-3 | 130 | 66 | 2.0 | 68 | 52.3 | 45 | 19 | 26 | 6 | 14 | 7.0 | 2 | 3.0 |
| *T. pediculiformis* pop. 1 | Tp1-4 | 138 | 52 | 2.7 | 78 | 56.5 | 45 | 22 | 29 | 6 | 8 | 9.5 | n.a. | n.a. |
| *T. pediculiformis* pop. 1 | Tp1-5 | 111 | 26 | 4.3 | 55 | 49.5 | 44 | 20 | 30 | 6 | 16 | 4.0 | 2 | 3.0 |
| *T. pediculiformis* pop. 1 | Tp1-6 | 149 | 38 | 3.9 | 67 | 45.0 | 43 | 18 | 25 | 6 | 16 | 8.0 | 2 | 2.9 |
| *T. pediculiformis* pop. 1 | Tp1-7 | 127 | 30 | 4.2 | 65 | 51.2 | 45 | 17 | 25 | 6 | 13 | 6.0 | 2 | 3.0 |
| *T. pediculiformis* pop. 1 | Tp1-8 | 142 | 42 | 3.4 | 70 | 49.3 | 43 | 16 | 23 | 6 | 15 | 6.0 | 2 | 3.0 |
| *T. pediculiformis* pop. 1 | Tp1-9 | 148 | 47 | 3.1 | 67 | 45.3 | 43 | 16 | 23 | 6 | 16 | 6.0 | 2 | 3.0 |
| *T. pediculiformis* pop. 1 | Tp1-10 | 135 | 47 | 2.9 | 65 | 48.1 | 45 | 18 | 25 | 6 | 16 | 6.0 | 2 | 2.8 |
| *T. pediculiformis* pop. 1 | Tp1-11 | 160 | 49 | 3.3 | 70 | 43.8 | 42 | 18 | 26 | 6 | 14 | 5.5 | 2 | 3.5 |
| *T. pediculiformis* pop. 1 | Tp1-12 | 135 | 47 | 2.9 | 65 | 48.1 | 42 | 18 | 25 | 6 | 7 | 6.0 | 2 | 2.8 |
| *T. pediculiformis* pop. 3 | Tp3-1 | 110.9 | 45.8 | 2.4 | 44.5 | 40.1 | 41 | 19 | 26 | 6 | 12 | 6.4 | 3 | 1.8 |
| *T. pediculiformis* pop. 3 | Tp3-2 | 139.4 | 55.4 | 2.5 | 83.5 | 59.9 | 44 | 19 | 26 | 6 | 16 | 6.3 | 2 | 2.2 |
| *T. pediculiformis* pop. 3 | Tp3-3 | 108.0 | 48.3 | 2.2 | 48.6 | 45.0 | 39 | 19 | 23 | 6 | 19 | 10.8 | 2 | 2.2 |
| *T. pediculiformis* pop. 3 | Tp3-4 | 122.3 | 70.2 | 1.7 | 69.5 | 56.8 | 36 | 18 | 26 | 6 | 13 | 5.7 | 1 | 2.2 |
| *T. pediculiformis* pop. 3 | Tp3-5 | 126.5 | 58.5 | 2.2 | 69.4 | 54.9 | 39 | 20 | 24 | 6 | 16 | 5.4 | 3 | 2.6 |
| *T. pediculiformis* pop. 3 | Tp3-6 | 161.9 | 82.5 | 2.0 | 72.4 | 44.7 | 43 | 22 | 25 | 6 | 16 | 4.6 | 2 | 2.1 |
| *T. pediculiformis* pop. 3 | Tp3-7 | 124.6 | 55.5 | 2.2 | 59.8 | 48.0 | 44 | 19 | 26 | 6 | 14 | 7.8 | 2 | 1.7 |
| *T. pediculiformis* pop. 3 | Tp3-8 | 130.3 | 55.1 | 2.4 | 66.3 | 50.9 | 41 | 17 | 25 | 6 | 18 | 9.1 | 4 | 2.4 |
| *T. pediculiformis* pop. 3 | Tp3-9 | 145.9 | 73.7 | 2.0 | 67.2 | 46.1 | 51 | 19 | 23 | 6 | 28 | 9.0 | 3 | 2.8 |
| *T. pediculiformis* pop. 3 | Tp3-10 | 131.6 | 50.7 | 2.6 | 54.6 | 41.5 | 43 | 18 | 24 | 6 | 26 | 7.2 | 1 | 2.5 |
| *T. pediculiformis* pop. 3 | Tp3-11 | 120.8 | 57.9 | 2.1 | 62.8 | 52.0 | 42 | 17 | 25 | 6 | 23 | 6.8 | 3 | 2.8 |
| *T. pediculiformis* pop. 3 | Tp3-12 | 117.8 | 55.9 | 2.1 | 62.0 | 52.6 | 53 | 22 | 33 | 6 | 26 | 5.7 | 4 | 2.7 |
| *T. pediculiformis* pop. 3 | Tp3-13 | 99.0 | 42.6 | 2.3 | 48.5 | 49.0 | 54 | 20 | 29 | 6 | 18 | 6.8 | n.a. | n.a. |
| *T. pediculiformis* pop. 3 | Tp3-14 | 131.4 | 67.4 | 1.9 | 53.9 | 41.0 | 54 | 26 | 35 | 6 | 32 | 9.0 | n.a. | n.a. |
| *T. pediculiformis* pop. 3 | Tp3-15 | 86.0 | 55.4 | 1.6 | 40.0 | 46.5 | 52 | 25 | 29 | 6 | 26 | 8.9 | n.a. | n.a. |
| *T. pediculiformis* pop. 3 | Tp3-16 | 130.0 | 80.7 | 1.6 | 70.2 | 54.0 | 49 | n.a. | n.a. | 6 | n.a. | 8.6 | n.a. | n.a. |

*Measurements in μm.*

*AM, adoral membranelles; MA, macronucleus; MI, micronucleus; n.a., data not available.*

**SUPPLEMENTARY TABLE S2 |** List of taxa with GenBank accession numbers of corresponding SSU rDNA, ITS1-5.8S-ITS2, and LSU rDNA sequences included in multi-gene phylogenetic analyses.

| **Taxon** | **SSU rDNA** | **ITS-5.8S-ITS2** | **LSU rDNA** |
| --- | --- | --- | --- |
| *Amphisiella* aff. *milnei* | KU594635 | KU594624 | KU594643 |
| *Amphisiella annulata* | DQ832260 | KU594625 | KU594644 |
| *Amphisiella candida* | JX461344 | KU594620 | KU594620 |
| *Amphisiella milnei* | FJ870072 | KU594626 | KU594645 |
| *Amphisiella pulchra* | JX461343 | KU594621 | KU594621 |
| *Amphisiella sinica* | FJ870073 | KU594627 | KU594646 |
| *Apodiophrys ovalis* | GU477634 | JF694069 | JF694045 |
| *Bakuella granulifera* | KJ958489 | KJ958522 | KJ958522 |
| *Bergeriella ovata* | FJ754026 | GQ246479 | JQ424856 |
| *Caudikeronopsis marina* | KR612270 | KX099175 | KX099175 |
| *Cyrtohymena citrina* | AF508755 | AF508755 | AF508755 |
| *Diaxonella trimarginata* | JQ424833 | JQ424860 | JQ424837 |
| *Diophrys scutum* | JF694040 | JF694073 | JF694054 |
| *Engelmanniella mobilis* | AF508757 | AF508757 | AF508757 |
| *Gastrostyla steinii* | AF508758 | AF508758 | AF508758 |
| *Gonostomum affine* | KU594638 | KU594628 | KU594647 |
| *Gonostomum kuehnelti* | MT247885 | MT247888 | MT247892 |
| *Gonostomum sinicum* | KY475614 | MT233441 | MT233439 |
| *Gonostomum strenuum* | KU594639 | KU594629 | KU594648 |
| *Halteria grandinella* | AF508759 | AF508759 | AF508759 |
| *Hemiamphisiella terricola qingdaoensis* | KM222091 | KM222032 | KM222120 |
| *Hemigastrostyla enigmatica* pop. 1 | KU594640 | KU594630 | KU594649 |
| *Hemigastrostyla enigmatica* pop. 2 | FJ870096 | KM222035 | KM222124 |
| *Heterokeronopsis pulchra* | JQ083600 | KJ697764 | KJ697764 |
| *Heterourosomoida longa* | AF508763 | AF508763 | AF508763 |
| *Holosticha bradburyae* | FJ775716 | KF306381 | KF306400 |
| *Holosticha diademata* | KF306396 | KF306391 | KF306412 |
| *Holosticha heterofoissneri* | KM222094 | KM222040 | KM222132 |
| *Laurentiella strenua* | JX893368 | AJ286807 | HM122019 |
| *Metaurostylopsis cheni* | FJ775720 | JQ424862 | JQ424843 |
| *Monocoronella carnea* | FJ775726 | JQ424870 | JQ424855 |
| *Neourostylopsis flava* | KR013238 | KY874010 | KY874010 |
| *Nothoholosticha fasciola* | FJ377548 | FJ858212 | JQ424846 |
| *Oxytricha granulifera* | AF508762 | AF508762 | AF508762 |
| *Parabirojimia multinucleata* | FJ156104 | GQ246482 | KM222129 |
| *Paradiophrys zhangi* | FJ870076 | JF694077 | JF694060 |
| *Paraurostyla viridis* | AF508766 | AF508766 | AF508766 |
| *Paraurostyla weissei* | AF508767 | AF508767 | AF508767 |
| *Paruroleptus lepisma* | AF508765 | AF508765 | AF508765 |
| *Pleurotricha lanceolata* | AF508768 | AF508768 | AF508768 |
| *Protogastrostyla pulchra* | KU594641 | KU594631 | KU594650 |
| *Psammomitra retractilis* | EF486865 | GQ246483 | KF306405 |
| *Pseudokeronopsis carnae* | KU663901 | KU663903 | JQ424836 |
| *Pseudourostyla cristata* | FJ598608 | GQ246486 | JQ424848 |
| *Spirotrachelostyla tani* | FJ870093 | KU594632 | KU594651 |
| **Taxon** | **SSU rDNA** | **ITS-5.8S-ITS2** | **LSU rDNA** |
| *Sterkiella histriomuscorum* | FJ545743 | FJ545743 | FJ545743 |
| *Sterkiella nova* | AF508771 | AF508771 | AF508771 |
| *Strongylidium orientale* | KC153532 | KM222034 | KM222122 |
| *Stylonychia lemnae* | AF508773 | AF508773 | AF508773 |
| *Stylonychia mytilus* | AF508774 | AF508774 | AF508774 |
| *Thigmokeronopsis rubra* | KX138656 | KX099221 | KX099220 |
| *Trachelostyla multinucleata* spec. nov. | MZ856308 | MZ856304 | MZ856306 |
| *Trachelostyla pediculiformis* pop. 1 | MZ856309 | MZ856305 | MZ856307 |
| *Trachelostyla pediculiformis* type pop. | DQ057346 | KU594633 | KU594652 |
| *Trachelostyla pediculiformis* pop. 3^*^ | KU594642 | KU594634 | KU594653 |
| *Uroleptus gallina* | AF508779 | AF508779 | AF508779 |
| *Uroleptus piscis* | AF508780 | AF508780 | AF508780 |
| *Uronychia multicirrus* | EU267929 | JF694078 | JF694061 |

* Misidentified material, which is from an undescribed isolate in Huang et al. (2016).

**SUPPLEMENTARY TABLE S3 |** Characterization and evolutionary models selected under the Bayesian information criterion by the IQ-Tree program for the two datasets analysed.

| **Dataset** | **Marker** | **No. of chars** | **No. of taxa** | **Model** | **A** | **C** | **G** | **T** | **[AC]** | **[AG]** | **[AT]** | **[CG]** | **[CT]** | **[GT]** | **I** | **Γ** |
| --- | --- | --- | --- | --- | --- | --- | --- | --- | --- | --- | --- | --- | --- | --- | --- | --- |
| #1 | 18S | 1‒1798 | 94 | TIM2 | 0.2680 | 0.1974 | 0.2561 | 0.2785 | 1.4358 | 2.7815 | 1.4358 | 1.0000 | 6.3900 | 1.0000 | 0.5303 | 0.4805 |
| #2 | 18S | 1‒1798 | 58 | TIM2 | 0.2682 | 0.1966 | 0.2558 | 0.2794 | 1.4481 | 2.8555 | 1.4481 | 1.0000 | 6.2770 | 1.0000 | 0.5666 | 0.5169 |
|  | ITS-5.8S | 1799‒2260 | 58 | SYM | 0.2500 | 0.2500 | 0.2500 | 0.2500 | 2.1955 | 4.5331 | 3.3158 | 0.6291 | 8.0153 | 1.0000 | – | 0.4114 |
|  | 28S | 2061‒3678 | 58 | TIM3e | 0.2500 | 0.2500 | 0.2500 | 0.2500 | 0.5420 | 1.7853 | 1.0000 | 0.5420 | 2.8240 | 1.0000 | 0.2707 | 0.5564 |

*Base frequencies (A, C, G, T), rate substitution matrix ([AC], [AG], [AT], [CG], [CT], [GT]), proportion of invariable sites (I), gamma distribution shape parameter (Γ).*

**SUPPLEMENTARY TABLE S4** **|** Numbers of unmatched nucleotides (above diagonal) and CBCs (below diagonal) in the ITS2 molecules of the family Trachelostylidae.

| Species | **1.** | **2.** | **3.** | **4.** | **5.** | **6.** |
| --- | --- | --- | --- | --- | --- | --- |
| **1.** *T. multinucleata* spec. nov. (MZ856304) |  | 21 | 21 | 23 | 21 | 25 |
| **2.** *T. pediculiformis* neotype pop. (KU594633) | 0 |  | 0 | 8 | 32 | 21 |
| **3.** *T. pediculiformis* pop. 1 (MZ856305) | 0 | 0 |  | 8 | 32 | 21 |
| **4.** *T. pediculiformis* pop. 2 (KM222038) | 0 | 0 | 0 |  | 28 | 24 |
| **5.** *T. pediculiformis* pop. 3 (KU594634) | 0 | 1 | 1 | 0 |  | 32 |
| **6.** *S. tani* (KU594632) | 0 | 1 | 1 | 0 | 0 |  |

**Reference alignment of the 18S rRNA gene**

**for detection of diagnostic molecular characters**

**>Trachelostyla_multinucleata_spec_nov**

TACATGGATAACCGTGGTAATTCTAGAGCTAATACATGCTGTTGTGCCTGACTTTTGGAA

GGGCTGTATTTATTAGATAACAAACCAATATTCCTTGTGTCTATTGTGGTGATTCATAGT

AACTGATCGAATCGCATGGACTTTGTCCGCGATAAATCATTCAAGTTTCTGCCCCATCAG

CTTTCGATGGTAGTGTATTGGACTACCATGGCTTTCACGGGTAACGGAGGATTAGGGTTC

GATTCCGGAGAGGGAGCCTGAGAAACGGCTACCACATCTACGGAAGGCAGCAGGCGCGTA

AATTACCCAATCCTGACTCAGGGAGGTAGTGACAAGAAATAACGGACCGAAGCTTTACGT

TTCGGGATCGCAATGGGTACAACTTAAACCCCTTAACGAGGATCAATTGGAGGGCAAGTC

TGGTGCCAGCAGCCGCGGTAATTCCAGCTCCAATAGCGTATATTAAAGTTGTTGCAGTTA

AAAAGCTCGTAGTTGGACCTCTGGGAGGGCGCCAATGTCCGCTGATGCGTGCGCAGCGGC

GCCCTTCCATCCTTCTGTTAACGTTTCTCGGTATTCAGTTACTGGTTTCGGGCTCAGATA

TTTTACCTTGAGAAAATTAGAGTGTTTCAGGCAGGCTTGCGCCGGAATACATTAGCATGG

AATAATAGAATAGGACTTTAGTCTCTCTTGTTGGTTTAGGGACTGAAGTAATGATTAATA

GGGATAGTTGGGGGCATTAGTATTTAATTGTCAGAGGTAAAATTCTCGGATTTGTTAAAG

ACTAACTTATGCGAAAGCATTTGCCAAGGATGTTTTCATTAATCAAGAACGAAAGTTAGG

GGATCCAAGACGATCAGATACCGTCCTAGTCTTAACCATAAACTATGCCGACTAGGGATC

GGAGGCGTGCAATTATCTGCCTTCGGCACCTTATGAGAAATCAAAGTCTTTGGGTTCTGG

GGGGAGTATGGTCGCAAGGCTGAAACTTAAAGGAATTGACGGAAGGGCACCACCAGGAGT

GGAGCTTGCGGCTCAATTTGACTCAACACGGGAAAACTTACCAGGTCCAGACATAGTGAG

GATTGACAGATTGATAGCTCTTTCTTGATTCTATGGGTGGTGGTGCATGGCCGTTCTTAG

TTGGTGGAGTGATTTGTCTGGTTAATTCCGTTAACGAACGAGACCTTAGCCTGCTAACTA

GTCGATTCAATTCCGATTGACTTCGACTTCTTAGAGGGACTTTGTGGCCAAACACAAGGA

AGTTTGAGGCAATAACAGGTCTGTGATGCCCTTAGATGTCCTGGGCCGCACGCGTGCTAC

ACTGACGCATGCAGCGAGTACTTCCC-AGCTCCGTGAGGCAGCTGGTAATCAGCAATATG

CGTCGTGATGGGGATAGATCTTTGGAATTCTGGATCTTGAACGAGGAATTCCTAGTAAGC

GCAAGTCATTAGCTTGCGCTGATTAAGTCCCTGCCCTTTGTACACACCGCCCGTCGCTCC

TACCGATTTCGAGTGATCCGGTGAACCTTTTGGACCGCTGGTGCCTCGTGTGCTGCGCGG

GAAATCAAGTAAACCATATCACTTAGAGGAAGG

**>Trachelostyla_pediculiformis_pop1**

TACATGGATAACCGTGGTAATTCTAGAGCTAATACATGCTGGTTTGCCTGACTTCTTGAA

GGGCTGTATTTATTAGATAACGAACCAATATTCCTTGTGTCTATTGTGGTGACTCATAGT

AACTGATCGAATCGCATGGACCTTGTCCGCGATAAATCATTCAAGTTTCTGCCCCATCAG

CTTTCGATGGTAGTGTATTGGACTACCATGGCTTTCACGGGTAACGGAGGATTAGGGTTC

GATTCCGGAGAGGGAGCCTGAGAAACGGCTACCACATCTACGGAAGGCAGCAGGCGCGTA

AATTACCCAATCCTGACTCAGGGAGGTAGTGACAAGAAATAACGGACCGAAGCATCTTGT

TTCGGGATTGCAATGGGTACAATTTAAACCCCTTAACGAGGATCAATTGGAGGGCAAGTC

TGGTGCCAGCAGCCGCGGTAATTCCAGCTCCAATAGCGTATATTAAAGTTGTTGCAGTTA

AAAAGCTCGTAGTTGGACTTCTGGGAGGGCGCCAATGTCCGCTGTTGCGTGTGCAGCGGC

TCCCTTCCATCCTTCTGTTAACGTTTCTTGGTATTCAGTTACTGGTTTCGGGCTCAGATA

TTTTACCTTGAGAAAATTAGAGTGTTTCAGGCAGGCTTGCGCCGGAATACATTAGCATGG

AATAATAGAATAGGACTTTAGTCTCTCTTGTTGGTTTAGGGACTGAAGTAATGATTAATA

GGGATAGTTGGGGGCATTAGTATTTAATTGTCAGAGGTAAAATTCTCGGATTTGTTAAAG

ACTAACTTATGCGAAAGCATTTGCCAAGGATGTTTTCATTAATCAAGAACGAAAGTTAGG

GGATCAAAGACGATCAGATACCGTCCTAGTCTTAACCATAAACTATGCCGACTAGGGATC

GGAGGCGTG-ACTTATCCGCCTTCGGCACCTTATGAGAAATCAAAGTCTTTGGGTTCTGG

GGGGAGTATGGTCGCAAGGCTGAAACTTAAAGGAATTGACGGAAGGGCACCACCAGGCGT

GGAGCTTGCGGCTCAATTTGACTCAACACGGGAAAACTTACCAGGTCCAGACATAGTGAG

GATTGACAGATTGATAGCTCTTTCTTGATTCTATGGGTGGTGGTGCATGGCCGTTCTTAG

TTGGTGGAGTGATTTGTCTGGTTAATTCCGTTAACGAACGAGACCTTAGCCTGCTAACTA

GTCGATTCAATCCTGATTGGCTTCGACTTCTTAGAGGGACTTTGTGACTAAACACAAGGA

AGTTTGAGGCAATAACAGGTCTGTGATGCCCTTAGATGTCCTGGGCCGCACGCGTGCTAC

ACTGACGCATGCAGCGAGCACTTCCCCGGCTCCGTGAGGCAGCCGGTAATCAGCAATATG

CGTCGTGATGGGGATAGATCTTTGGAATTCTGGATCTTGAACGAGGAATGCCTAGTAAGC

GCAAGTCATTAGCTTGCGCTGACTAAGTCCCTGCCCTTTGTACACACCGCCCGTCGCTCC

TACCGATTTCGAGTGCTCCGGTGAACCTTTTGGACCGCTGGCACCTCGTGTGCTGCGCGG

GAAATCAAGTAAACCATATCACTTAGAGGAAGG

**>Trachelostyla_pediculiformis_neotype_pop**

TACATGGATAACCGTGGTAATTCTAGAGCTAATACATGCTGGTTTGCCTGACTTCTTGAA

GGGCTGTATTTATTAGATAACGAACCAATATTCCTTGTGTCTATTGTGGTGACTCATAGT

AACTGATCGAATCGCATGGACCTTGTCCGCGATAAATCATTCAAGTTTCTGCCCCATCAG

CTTTCGATGGTAGTGTATTGGACTACCATGGCTTTCACGGGTAACGGAGGATTAGGGTTC

GATTCCGGAGAGGGAGCCTGAGAAACGGCTACCACATCTACGGAAGGCAGCAGGCGCGTA

AATTACCCAATCCTGACTCAGGGAGGTAGTGACAAGAAATAACGGACCGAAGCATCTTGT

TTCGGGATTGCAATGGGTACAATTTAAACCCCTTAACGAGGATCAATTGGAGGGCAAGTC

TGGTGCCAGCAGCCGCGGTAATTCCAGCTCCAATAGCGTATATTAAAGTTGTTGCAGTTA

AAAAGCTCGTAGTTGGACTTCTGGGAGGGCGCCAATGTCCGCTGTTGCGTGTGCAGCGGC

TCCCTTCCATCCTTCTGTTAACGTTTCTTGGTATTCAGTTACTGGTTTCGGGCTCAGATA

TTTTACCTTGAGAAAATTAGAGTGTTTCAGGCAGGCTTGCGCCGGAATACATTAGCATGG

AATAATAGAATAGGACTTTAGTCTCTCTTGTTGGTTTAGGGACTGAAGTAATGATTAATA

GGGATAGTTGGGGGCATTAGTATTTAATTGTCAGAGGTAAAATTCTCGGATTTGTTAAAG

ACTAACTTATGCGAAAGCATTTGCCAAGGATGTTTTCATTAATCAAGAACGAAAGTTAGG

GGATCAGAGACGATCAGATACCGTCCTAGTCTTAACCATAAACTATGCCGACTAGGGATC

GGAGGCGTG-ACTTATCCGCCTTCGGCACCTTATGAGAAATCAAAGTCTTTGGGTTCTGG

GGGGAGTATGGTCGCAAGGCTGAAACTTAAAGGAATTGACGGAAGGGCACCACCAGGCGT

GGAGCTTGCGGCTCAATTTGACTCAACACGGGAAAACTTACCAGGTCCAGACATAGTGAG

GATTGACAGATTGATAGCTCTTTCTTGATTCTATGGGTGGTGGTGCATGGCCGTTCTTAG

TTGGTGGAGTGATTTGTCTGGTTAATTCCGTTAACGAACGAGACCTTAGCCTGCTAACTA

GTCGATTCAATCCTGATTGGCTTCGACTTCTTAGAGGGACTTTGTGACTAAACACAAGGA

AGTTTGAGGCAATAACAGGTCTGTGATGCCCTTAGATGTCCTGGGCCGCACGCGTGCTAC

ACTGACGCATGCAGCGAGCACTTCCCCGGCTCCGTGAGGCAGCCGGTAATCAGCAATATG

CGTCGTGATGGGGATAGATCTTTGGAATTCTGGATCTTGAACGAGGAATGCCTAGTAAGC

GCAAGTCATTAGCTTGCGCTGACTAAGTCCCTGCCCTTTGTACACACCGCCCGTCGCTCC

TACCGATTTCGAGTGCTCCGGTGAACCTTTTGGACCGCTGGCACCTCGTGTGCTGCGCGG

GAAATCAAGTAAACCATATCACTTAGAGGAAGG

**> Trachelostyla_pediculiformis_pop3**

TACATGGATAACCGTGGTAATTCTAGAGCTAATACATGCTGGTTTGCCTGACTTTTGGAA

GGGCTGTATTTATTAGATAACAAACCAATATTCCTTGTGCCTATTGTGGTGATTCATAGT

AACTGATCGAATCGCATGGACCTTGTCCGCGATAAATCATTCAAGTTTCTGCCCCATCAG

CTTTCGATGGTAGTGTATTGGACTACCATGGCTTTCACGGGTAACGGAGGATTAGGGTTC

GATTCCGGAGAGGGAGCCTGAGAAACGGCTACCACATCTACGGAAGGCAGCAGGCGCGTA

AATTACCCAATCCTGTCTCAGGGAGGTAGTGACAAGAAATAACGGACCGAAGCTTCATGT

TTCGGGATTGCAATGGGTACAATTTAAACCCCTTAACGAGGATCAATTGGAGGGCAAGTC

TGGTGCCAGCAGCCGCGGTAATTCCAGCTCCAATAGCGTATATTAAAGTTGTTGCAGTTA

AAAAGCTCGTAGTTGGACTTCTGGGAGGGTGCCAATGTCCACTGTTGTGAGAGCAGCGGG

GCCCTTCCATCCTTCTGTTAACGTTTCTTGGTATTCATTTACTGGTTTCGGGCTCAGATA

TTTTACCTTGAGAAAATTAGAGTGTTTCAGGCAGGCTTGCGCCGGAATACATTAGCATGG

AATAATAGAATAGGACTTCTCTCTCTCTTGTTGGTTTAGGGACTGAAGTAATGATTAATA

GGGATAGTTGGGGGCATTAGTATTTAATTGTCAGAGGTAAAATTCTCGGATTTGTTAAAG

ACTAACTTATGCGAAAGCATTTGCCAAGGATGTTTTCGTTAATCAAGAACGAAAGTTAGG

GGATCAAAGACGATCAGATACCGTCCTAGTCTTAACCATAAACTATGCCGACTAGGGATC

GGAGGCGCGTTAT-ATCCGCCTTCGGCACCTTATGAGAAATCAAAGTCTTTGGGTTCTGG

GGGGAGTATGGTCGCAAGGCTGAAACTTAAAGGAATTGACGGAAGGGCACCACCAGGAGT

GGAGCTTGCGGCTCAATTTGACTCAACACGGGAAAACTTACCAGGTCCAGACATAGTGAG

GATTGACAGATTGATAGCTCTTTCTTGATTCTATGGGTGGTGGTGCATGGCCGTTCTTAG

TTGGTGGAGTGATTTGTCTGGTTAATTCCGTTAACGAACGAGACCTTAGCCTGCTAACTA

GTCGATTCAATTCTGATTGACTTTGACTTCTTAGAGGGACTTTGTGGCTAAACACAAGGA

AGTTTGAGGCAATAACAGGTCTGTGATGCCCTTAGATGTCCTGGGCCGCACGCGTGCTAC

ACTGACGCATGCAGCGAGTACTTCCC-AGCTCCGTGAGGCAGCTGGTAATCAGCAATATG

CGTCGTGATGGGGATAGATCTTTGGAATTCTGGATCTTGAACGAGGAATTCCTAGTAAGC

GCAAGTCATTAGCTTGCGCTGATTAAGTCCCTGCCCTTTGTACACACCGCCCGTCGCTCC

TACCGATTTCGAGTGATCCGGTGAACCTTTTGGACCGCTGGTACCTCGTGTGCTGTGCGG

GAAATCAAGTAAACCATATCACTTAGAGGAAGG

**>Spirotrachelostyla_tani**

TACATGGATAACCGTGGTAATTCTAGAGCTAATACATGCTGGTGTGCCCGACTCAC-GAA

GGGCTGTATTTATTAGATAACAAACCAATATTCCTTGTGTCTATTGTGATGACTCATAGT

AACTGATCGAATCGCATGGATTTTGTCCGCGATAAATCATTCAAGTTTCTGCCCCATCAG

CTTTCGATGGTAGTGTATTGGACTACCACGGCTTTCACGGGTAACGGAGGATTAGGGTTC

GATTCCGGAGAGGGAGCCTGAGAAACGGCTACCACATCTACGGAAGGCAGCAGGCGCGTA

AATTACCCAATCCTGACTCAGGGAGGTAGTGACAAGAAATAACGGACCGAAGCTTACTGT

TTCGGGATTGCAATGGGTACAATATAAACCCCTTAGCGAGGATCAATTGGAGGGCAAGTC

TGGTGCCAGCAGCCGCGGTAATTCCAGCTCCAATAGCGTATATTAAAGTTGTTGCAGTTA

AAAAGCTCGTAGTTGGATTTCTGGGAGGGTGCCAATGTCCGCTGAGGCGAGTGCAGCCGC

GCCCTTCCATCCTTCTGTTAACGTTTCTCGGTATTCAGTTACTGGTTTCGGGCTCAGATA

TTTTACCTTGAGAAAATTAGAGTGTTTCAGGCAGGCTTGCGCCGGAATACATTAGCATGG

AATAATTGAAAAGGACTTTAGTCTCCCTTGTTGGTTTAGGGACTGAAGTAATGATTAATA

GGGATAGTTGGGGGCATTAGTATTTAATTGTCAGAGGTAAAATTCTCGGATTTGTTAAAG

ACTAACTTATGCGAAAGCATTTGCCAAGGATGTTTTCATTAATCAAGAACGAAAGTTAGG

GGATCAAAGACGATCAGATACCGTCCTAGTCTTAACCATAAACTATGCCGACTAGGGATC

GGAGGCGTGGCAT-ATCCGCCTTCGGCACCTTATGAGAAATCAAAGTCTTTGGGTTCTGG

GGGGAGTATGGTCGCAAGGCTGAAACTTAAAGGAATTGACGGAAGGGCACCACCAGGAGT

GGAGCTTGCGGCTCAATTTGACTCAACACGGGAAAACTTACCAGGTCCAGACATAGTGAG

GATTGACAGATTGATGGCTCTTTCTTGATTCTATGGGTGGTGGTGCATGGCCGTTCTTAG

TTGGTGGAGTGATTTGTCTGGTTAATTCCGTTAACGAACGAGACCTTAGCCTGCTAACTA

GTCGATTCAATCCCGATTGACTTCGACTTCTTAGAGGGACTTTGTGGCCAAACACAAGGA

AGTTTGAGGCAATAACAGGTCTGTGATGCCCTTAGATGTCCTGGGCCGCACGCGTGCTAC

ACTGACGCATGCAGCGAGTACTTCCC-AGCTCCGTGAGGCAGCTGGTAATCAAAAATATG

CGTCGTGATGGGGATAGATCTTTGGAATTCTGGATCTTGAACGAGGAATTCCTAGTAAGC

GCAAGTCATTAGCTTGCGCTGACTAAGTCCCTGCCCTTTGTACACACCGCCCGTCGCTCC

TACCGATTTCGAGTGATTCGGTGAACCTTTTGGACCGCTGGCTCCTCG-GAGCTGCGCGG

GAAATCAAGTAAACCACATCACTTAGAGGAAGG

**Reference alignment of the ITS1-5.8S-ITS2 region**

**for detection of diagnostic molecular characters**

**>Trachelostyla_multinucleata_spec_nov**

CACTAATCCACAAACCTAAACTGAACCTTTGTACAGTGCTGCTGACGCCTAGTGCGCGTG

GTACTGTCTCAAAACTAACAAAAGGAACTAACTAAGTCCTTACTA-AAACCAAATTTTCA

ACGATGGATATCTTGGTTCCCGCAACGATGAAGAACGCAGCGAAGTGCGATAAGCAATGC

GAATTGCAGAACCGTGAGTCATCAGATTTTTGAACGCAACTGGCGCCGATTGGTATCCCA

GTCGGCATGCTTGTTTCAGTGTCTCGCGCACTCACCAATATCTTAATGCGAGAGATACTC

TTCTCTCGTTAAGCATGAAGGCGCTACTGCGCTCTGCAGCGGGTCTTC----CCGCACTC

AATGCAGCCGTCACATTCTTTAATGTGAACCTCATTGAGGTAGAGGCAGA-GGCGTCTAG

TCTTCCAACCTTTTTTGCATCTGAAATCAAGCAGGAGTAC

**>Trachelostyla_pediculiformis_pop1**

CACTAATCCACAAACCTAAACCGAACCTTTGCACAGTGCTGCCGACGCCTCGAGCGCGTG

GTGCTGTCTCAAACCTAACTAAAGGAACTAACTAAGTCCTTACCATAAACCAAATTTTCA

ACGATGGATATCTTGGTTCCCGCAACGATGAAGAACGCAGCGAAGTGCGATAAGCAATGC

GAATTGCAGAACCGTGAGTCATCAGATTTTTGAACGCAACTGGCGCCGATGGGTATCCCC

GTCGGCATGCTTGTTTCAGTGTCTTGCGCACTCACCAAAATCTTAATGCGAGAGATACCC

TTCTCTTGCCAAGCATGAAGACGCTACTGCGCTCTGCAGCGGGTCCTCGGACTCGCACTC

AATGCAGCAGTCACATTCTTCAATGTGAACCTCATTGAGGCAGGGGCTGA-GGCGTCTAG

TCTTCCAACCTTCTCTGTATCTGAAATCAAGCAGGATTAC

**>Trachelostyla_pediculiformis_neotype_pop**

CACTAATCCACAAACCTAAACCGAACCTTTGCACAGTGCTGCCGACGCCTCGAGCGCGTG

GTGCTGTCTCAAACCTAACTAAAGGAACTAACTAAGTCCTTACCATAAACCAAATTTTCA

ACGATGGATATCTTGGTTCCCGCAACGATGAAGAACGCAGCGAAGTGCGATAAGCAATGC

GAATTGCAGAACCGTGAGTCATCAGATTTTTGAACGCAACTGGCGCCGATGGGTATCCCC

GTCGGCATGCTTGTTTCAGTGTCTTGCGCACTCACCAAAATCTTAATGCGAGAGATACCC

TTCTCTTGCCAAGCATGAAGACGCTACTGCGCTCTGCAGCGGGTCCTCGGACTCGCACTC

AATGCAGCAGTCACATTCTTCAATGTGAACCTCATTGAGGCAGGGGCTGA-GGCGTCTAG

TCTTCCAACCTTCTCTGTATCTGAAATCAAGCAGGATTAC

**>Trachelostyla_pediculiformis_pop2**

CACTAATCCACAAACCTAAACTGAACCTTTGCACAGTGCTGCCGACGCCTTGAGCGCGCG

GCGCTGTCTCAAACCTATCAAAAGGAACTAACTAAGTCTTTACCATAAACCAAATTTTCA

ACGATGGATATCTTGGTTCCCGCAACGATGAAGAACGCAGCGAAGTGCGATAAGCAATGC

GAATTGCAGAACCGTGAGTCATCAGATTTTTGAACGCAACTGGCGCCGATTGGCATCCCA

GTCGGCATGCTTGTTTCAGTGTCTTACGCACTCACCCAAATCTTAATGCGAGAGATGCTC

TTCTCTTGCTAAGTATGAAGGCGCTACTGCGCTCTGCAGCGGGTTCTCGGACTCGCACTC

AATGCAGCAGTCACATTCTTCAATGTGAACCTCATTGAGGCAGGGGCTGA-GGCGTCTAG

TCTTCCAACCTTCTCTGTATCTGAAATCAAGCAGGATTAC

**>Trachelostyla_pediculiformis_pop3**

CACTAATCCAAAAACCTAAACTGAACCTTTGCACAGTGCTGTTGATTCTTCCAGCGC-TG

G--CTATTCAACTCTCA--AAACTCAAGTAAATAAGCCTTTTACTTA---CAAATTTTCA

ACGATGGATATCTTGGTTCCCGTAACGATGAAGAACGCAGCGAAGTGCGATAAGCAATGC

GAATTGCAGAACCGTGAGTCATCAGATTTTTGAACGCAACTGGCGCCGATTGGCATCCCA

GTCGGCATGCTTGTTTCAGTGTCTTACGCAATCACCAAAATCTAAATGCGAGAGATGCTC

TTCTCTTGTTAAGCTCAAAGGCGCTACTGCGCTCTGCAGCGGTTTT------CCGCACTC

AATGCAGCAGTCACATTCTTTAATGTGAACCTCATTGAGGTAGAGGTAGC-GGCTCCTAG

TCTTTCAACCTTCTTTGCATCTGAAATCAAGCAGGAGTAC

**>Spirotrachelostyla_tani**

CACTAATCCACAAACCTAAAC-GAACCTTTGCACAGTGCTGCTGACGCCTTGAGCGCGCA

GCGCTGTCTCACAACTATCAAACGGAACTAACTAAGCCTTTAC-AAAAACCAAATTTTCA

ACGATGGATATCTTGGTTCCCGTAACGATGAAGAACGCAGCGAAGTGCGATAAGCAATGC

GAATTGCAGAACCGTGAGTCATCAGATTTTTGAACGCAACTGGCGCCGATTGGTACCCCG

GTCGGCATGCTTGTTTCAGTGTCTCGCAAACTCACCAAAATCTTAATGCGAGAGATACTC

TTCTCTCGTCAAGCTCGAAGGCGCTACTGCGCTCTGCAGCGGGT-CTCGG-CCCGCACTC

AATGCAGCAGTCACATTTCTCAATGTGAACCTCATTGAGGCAGGGGTGGTTGGCGCCTAG

TCTCCCAACCTTTTCTGTATCTGAAATCAAGCAGGATTAC

**Reference alignment of the 28S rRNA gene**

**for detection of diagnostic molecular characters**

**>Trachelostyla_multinucleata_spec_nov**

TCAGTAACGGCGAGTGAAGCGGCAAGAGCCCAACATGAAAATCTTCACGCATTTCGCGTG

ACGAATTGTAGGGTATAGAGGAGTTGCTCAACCGGCGTGGGCGCAGAAGTGCCTTAGAAC

AGGCGGCCAGAGAGGGTGACAGCCCCGTATGTGGTGCCCACACCTAACGAGGGACTTTTC

GAAGAGTCGGGTTGTTTTGTATTGCAGCCCTAAATGGGAGATAAACTTCTTCTAAGGCTA

AATATTTGCGGGAAACCGATAGCGAACAAGTACTGTGAAGGAAAGATGAAAAGAACTTTG

AAAAGAGAGTCAAAAGACTTGAAATCGTTGAGAAGGAAGCGGTAGAAATTTATTCTTCGG

CGGTGAACGGTGGCAGTTGTCTAACTTTATGCTGGCCTAGATACTTCATCGGTCTGGTTG

GTATCCAAGGGAAACGGCTGTCTTGTTCTTCGTCGTGAGGCAAAATGGAGTTGCTCGCCT

TCAGAGGCTTGGCAGGGCTGGCGCTATTCGTAGTGCACCCTGGCTGTG-CCGGAAGGTGA

GTGGCTTGAGGAGCTTCGTGCGATTTTGCCAAAATGGTTTTTACCGACCCGTCTTGAAAC

ACGGACCAAGGAGTCTAACATATATGCGAGTATGCTGGTGGAGAAACCAACATGCACAAC

TAACGTGAGCGATGCCAAGCTTT-GCAGCAGCATCGGCCGACCATGATTCTCTGATGAAA

GGATCGCGCAAGAGCATATCTGTTAGGACCCGAAAGATGGTGAACTATGCCTGAGCAGGG

TGAAGCCAGGGGAAACTCTGGTGGAGGCTCGTAGCGACACTGACGTGCAAATCGTTCGTC

TGACTTGGGTATAGGGGCGAAAGACTAATCGAACCATCTAGTAGCTGGTTCCCTCCGAAG

TTTCCCTCAGGATAGCAAGGACAATAATGCAGTTTTATTAGGTAAAGCGAATGATTAGAG

GCCTCGGGGATCCAGCATCCTCGACCTATTCTCAAACTTTAAATTGGTAAGAACCCTGGC

CTTCCTTAATTGAGGCGCAGGGGCTCAATGCGTGTCCTTAGTGGGCCATTTTTGGTAAGC

AGAACTGGCGATGAGGGATGCTCCTAACGTCGAGTTAAGGTGCCTAAATGCACGCTCATC

AG-ACACTACAAAGGGTGTTGGTTCATAAAGACAGCAGGACGGTGGCCATGGAAGTCGGA

ATCCGCTAAGGAGTGTGTAACAACTCACCTGCCGAATGAACTAGCCCCGAAAATGGATGG

CGCTTAAGCGTGCGACCGATACTCGACCATTGCGGCGAGAGTGAGGCTGCAATGAGTAGG

AGGGCGTGAGCGTTGTTGTGAAGCCTCTGACGTGAGTCTGGGTTGAACAGCGTTTAGTGC

GGATCTTGGTGGTAGTAGCAATTATTCAAATGAGAACTTTGAAGGCCGAAGTGGAGAAGG

TTTCCGTGAGAACAGCAATTGGTCACGGGTGACTCGATCCTAAGACATGGGGGAAATCCT

TGTTAAGTGCGCCGTATGGC---GCGAAGTCGAAAGGGAATGAGGTTAATATTCCTCAAG

CTGGATGTGGATATTGTATGGCAACATGAATGAGCTCAGAGACGCCAGCGTGAGCCTCTG

GAAGAGTTCTCTTTTCTTTTTAACAGATTAGCGACCTTGAAATTGGATTACCCAGAGCTA

AGGTTGTACATCTGGAAGAGCACCTCGCTTTTCGAGGTGTCAGGTGCGCTCACGATGGTC

CGTGAAAATCTGAGTGAGAGTC

**>Trachelostyla_pediculiformis_pop1**

CCAGTAACGGCGAGTGAAGCGGCTAGAGCCCAACATGAAAATCTTCACGCATTCAGCGTG

ACGAGTTGTAGGGTATAGGGGAGCTGCTCAACGGGCGTGGGCGCAGAAGTGCCTTAGAAC

AGGCGGCCAGAGAGGGTGACAGCCCCGTATGTGGTGCCCACACCTAACGAGCGACTTTCC

GAAGAGTCGGGTTGTTTTGTATTGCAGCCCTAAGTGGGAGATAAACTTCTTCTAAGGCTA

AATATTTGCGGGAAACCGATAGCGAACAAGTACTGTGAAGGAAAGATGAAAAGAACTTTG

AAAAGAGAGTCAAAAGACTTGAAATCGTTGAGAAGGAAGCGGTAGAAATTTATTCTTCGG

CGGTGAACGGTGGTAGTTGCCTAACTTCGCGCGTGGCTTGGTACGTCAATGGCCGGTCAT

GTGTTCAAGGGAAGCGGCTGCTTTGTTCTTCGTCGTGAGGCAAAATGGGGTTTCCATTAG

TCAGACTCGTCGCGAGGCTGGCCGGATTCGTCCGGCACCTCGCGGAAG-AGGGACCGGTG

GAGGCCTGAGGGGCTTCGTGCGATTTTGCCAAAATGGTTTTTACCGACCCGTCTTGAAAC

ACGGACCAAGGAGTCTAACATGTATGCGAGTATGCTGGTGGAAAAACCGACATGCACAAC

TAACGTGAACGATGCCAAGCGCAAGCAGCAGCATCGGCCGACCATGATTCTTTGATGAAA

GGATCGCGCAAGAGCATACCTGTTAGGACCCGAAAGATGGTGAACTATGCCTGAGCAGGG

TGAAGCCAGGGGAAACTCTGGTGGAGGCTCGTAGCGATACTGACGTGCAAATCGTTCGTC

TGACTTGGGTATAGGGGCGAAAGACTAATCGAACCATCTAGTAGCTGGTTCCCTCCGAAG

TTTCCCTTAGGATAGCAAGGACAATAATGCAGTTTTATTAGGTAAAGCGAATGATTAGAG

GCCTCGGGGGTCTAGCACCCTCGACCTATTCTCAAACTTTAAATTGGTAAGAACCCTGGC

CTTCCTTAATTGAGGCGCAGGGGCTCAATGCGTGTCCTTAGTGGGCCATTTTTGGTAAGC

AGAACTGGCGATGAGGGATGCTCCTAACGTCGAGTTAAGGTGCCGAAATGCACGCTCATC

AG-ACACTACAAAGGGTGTTGGTTCATAAAGACAGCAGGACGGTGGCCATGGAAGTCGGA

ACCCGCTAAGGAGTGTGTAACAACTCACCTGCCGAATGAACTAGCCCCGAAAATGGATGG

CGCTTAAGCGTGCGACCGATACTCGACCATTGCAGCGAGAGTGAGGCCGCAATGAGTAGG

AGGGCGTGAGCGTTGTTGTGAAGCCTCTGACGTGAGTCTGGGTTGAACAGCGTTTAGTGC

GGATCTTGGTGGTAGTAGCAATTATTCAAATGAGAACTTTGAAGGCCGAAGTGGAGAAGG

TTTCCGTGAGAACAGCAATTGGTCACGGGTGACTCGATCCTAAGACATGGGGGAAATCCT

TGTTAAGTGCGCCGTATGGC---GCGTAGTCGAAAGGGAAAGAGGTTAATATTCCTCTAG

CTGGATGTGGATATTGTGTGGCAACACTAGTGAGCTCAGAGACATCAGCGTGAGCCTCTG

GAAGAGTTATCTTTTCTTTTTAACAGACTAGCGACCTTGAAATTGGATTACCCAGAGCTA

AGGTTGTATGTCTGGAAGAGCACCTCGCTTTTCGAGGTGTCAGGTGCGCTCACGATGGTC

CTTGAAAATCTGAGTGAGAGTT

**>Trachelostyla_pediculiformis_neotype_pop**

CCAGTAACGGCGAGTGAAGCGGCTAGAGCCCAACATGAAAATCTTCACGCATTCAGCGTG

ACGAGTTGTAGGGTATAGGGGAGCTGCTCAACGGGCGTGGGCGCAGAAGTGCCTTAGAAC

AGGCGGCCAGAGAGGGTGACAGCCCCGTATGTGGTGCCCACACCTAACGAGCGACTTTCC

GAAGAGACGGGTTGTTTTGTATTGCAGCCCTAAGTGGGAGATAAACTTCTTCTAAGGCTA

AATATTTGCGGGAAACCGATAGCGAACAAGTACTGTGAAGGAAAGATGAAAAGAACTTTG

AAAAGAGAGTCAAAAGACTTGAAATCGTTGAGAAGGAAGCGGTAGAAATTTATTCTTCGG

CGGTGAACGGTGGTAGTTGCCTAACTTCGCGCGTGGCTTGGTACGTCAATGGCCGGTCAT

GTGTTCAAGGGAAGCGGCTGCTTTGTCCTTCGTCGTGAGGCAAAATGGGGTTTCCATTAG

TCAGACTCGTCGCGAGGCTGGCCGGATTCGTCCGGCACCTTGCGGAAG-AGGGACCGGTG

GAGGCCTGAGGGGCTTCGTGCGATTTTGCCAAAATGGTTTTTACCGACCCGTCTTGAAAC

ACGGACCAAGGAGTCTAACATGTATGCGAGTATGCTGGTGGAAAAACCGACATGCACAAC

TAACGTGAACGATGCCAAGCGCAAGCAGCAGCATCGGCCGACCATGATTCTTTGATGAAA

GGATCGCGCAAGAGCATACCTGTTAGGACCCGAAAGATGGTGAACTATGCCTGAGCAGGG

TGAAGCCAGGGGAAACTCTGGTGGAGGCTCGTAGCGATACTGACGTGCAAATCGTTCGTC

TGACTTGGGTATAGGGGCGAAAGACTAATCGAACCATCTAGTAGCTGGTTCCCCCCGAAG

TTTCCCTTAGGATAGCAAGGACAATAATGCAGTTTTATTAGGTAAAGCGAATGATTAGAG

GCCTCGGGGGTCTAGCACCCTCGACCTAT-CTCAAACTTTAAATTGGTAAGAACCCTGGC

CTTCCTTAATTGAGGCGCAGGGGCTCAATGCGTGTCCTTAGTGGGCCATTTTTGGTAAGC

AGAACTGGCGATGAGGGATGCTCCTAACGTCGAGTTAAGGTGCCGAAATGCACGCTCATC

AG-ACACTACAAAGGGTGTTGGTTCATAAAGACAGCAGGACGGTGGCCATGGAGGTCGGA

ACCCGCTAAGGAGTGTGTAACAACTCACCTGCCGAATGAACTAGCCCCGAAAATGGATGG

CGCTTAAGCGTGCGACCGATACTCGACCATTGCAGCGAGAGTGAGGCCGCAATGAGTAGG

AGGGCGTGAGCGTTGTTGTGAAGCCTCTGACGTGAGTCTGGGTTGAACAGCGTTTAGTGC

GGATCTTGGTGGTAGTAGCAATTATTCAAATGAGAACTTTGAAGGCCGAAGTGGAGAAGG

TTTCCGTGAGAACAGCAATTGGTCACGGGTGACTCGATCCTAAGACATGGGGGAAATCCT

TGTTAAGTGCGCCGTATGGC---GCGTAGTCGAAAGGGAAAGAGGTTAATATTCCTCTAG

CTGGATGTGGATATTGTGTGGCAACACTAGTGAGCTCAGAGACGCCAGCGTGAGCCTCTG

GAAGAGCTATCTTTTCTTTTTAACAGACTAGCGACCTTGAAATTGGATTACCCAGAGCTA

AGGTTGTATGTCTGGAAGAGCACCTCGCTTTTCGAGGTGTCAGGTGCGCTCACGATGGTC

CTTGAAAATCTGAGTGAGAGTT

**>Trachelostyla_pediculiformis_pop2**

CCAGTAACGGCGAGTGAAGCGGCTAGAGCCCAACATGAAAATCTCCACGCATTCCGTGTG

GCGAGTTGTAGGGTATAGGGAGGCTGCTCAACTGGCGTGCGCGCAGAAGTGCCTTAGAAC

AGGCGGCCATAGAGGGTGACAGCCCCGTATGTGGTGCGCACACCTAACGAGCGACTTTCC

GAAGAGTCGGGTTGTTTTGTATTGCAGCCCTAAGTGGGAGATAAACTTCTTCTAAGGCTA

AATATTTGCGGGAAACCGATAGCGAACAAGTACTGTGAAGGAAAGATGAAAAGAACTTTG

AAAAGAGAGTCAAAAGACTTGAAATCGTTGAGAAGGAAGCGGTAGAAATTTATTCTTCGG

CGGTGAACGGTGGAGGTTGCCTAACTTCGCGTGTGGCTTGGTACGTCAAAGGCCGGTCGT

GCGTTCAAGGGAAGTGGCCTCTTTGTTCTTCGTCGTGAGGCAAAATGGGGTTTCCATCGA

TCAGACTCGTTTCAAGGCTGGCAGGATCCGTCTTGCACCTTGAGGCAGTAGGGACTGGTG

GAGGCCTGAGGGGCTTCGTGCGATTTTGCCAAAATGGTTTTTACCGACCCGTCTTGAAAC

ACGGACCAAGGAGTCTAACATGTATGCGAGTATGCTGGTGGAAAAACCGACATGCACAAC

TAACGTGAACGATGCCAAGCGCAAGCAGCAGCATCGGCCGACCATGATTCTCTGATGAAA

GGATCGCGCAAGAGCATACCTGTTAGGACCCGAAAGATGGTGAACTATGCCTGAGCAGGG

TGAAGCCAGGGGAAACTCTGGTGGAGGCTCGTAGCGATACTGACGTGCAAATCGTTCGTC

TGACTTGGGTATAGGGGCGAAAGACTAATCGAACCATCTAGTAGCTGGTTCCCTCCGAAG

TTTCCCTCAGGATAGCAAGGACAATTATGCAGTTTTATTAGGTAAAGCGAATGATTAGAG

GCCTCGGGGATCTAGCATCCTCGACCTATTCTCAAACTTTAAATTGGTAAGAACCCTGGC

CTTCCTTAATTGAGGCGCAGGGGCTCAATGCGTGTCCTTAGTGGGCCATTTTTGGTAAGC

AGAACTGGCGATGAGGGTTGCTCCTAACGTCGAGTTAAGGTGCCGAAATGCACGCTCATC

AG-ACACTACAAAGGGTGTTGGTTCATAAAGACAGCAGGACGGTGGCCATGGAAGTCGGA

ATCCGCTAAGGAGTGTGTAACAACTCACCTGCCGAATGAACTAGCCCCGAAAATGGATGG

CGCTTAAGCGTGCGACCGATACTCGACCATTGCAGCGAGAGTGAGGCCGCAATGAGTAGG

AGGGCGTGAGCGTTGTTGTGAAGCCTCTGACGTGAGTCTGGGTTGAACAGCGTTTAGTGC

GGATCTTGGTGGTAGTAGCAATTATTCAAATGAGAACTTTGAAGGCCGAAGTGGAGAAGG

TTTCCGTGAGAACAGCAATTGGTCACGGGTGACTCGATCCTAAGACATGGGGGAAATCCT

TGTTAAGTGCGCCGTATGGC---GCGTAGTCGAAAGGGAATGAGGTTAATATTCCTCAAG

CTGGATGTGGATATTGTGTGGCAACACTAATGAGCTCAGAGACGCCAGCGTGAGCCTCTG

GAAGAGTTCTCTTTTCTTTTTAACAGACTAGCGACCTTGAAATTGGATTACCCAGAGCTA

AGGTTGTACGTCTGGAAGAGCACCTCGCTTTTCGAGGTGTCAGGTGCGCTCACGATGGTC

CGTGAAAATCTGAGTGAGAGTT

**>Trachelostyla_pediculiformis_pop3**

TCAGTAACGGCGAGTGAAGCGGCAAGAGCCCACCATGAAAATCTTCACGCATTTCGCGTG

ACGAGTTGTAGGGTAAAGGGTGGTTGCTCAACTGGCGTGGGCGCAGAAGTGCCTTAGAAC

AGGCGGCCAAAGAGGGTGACAGCCCCGTATGTGGTGCCCACACCTAACGAGTGATTGTCC

GAAGAGTCGGGTTGTTTTGTATTGCAGCCCTAATTGGGAGATAAACTTCTTCTAAGGCTA

AATACTTACGGGAAACCGATAGCGAACAAGTACTGTGAAGGAAAGATGAAAAGAACTTTG

AAAAGAGAGTCAAAAGACTTGAAATCGTTGAAAAGGAAGCGGTGGAAATTTATTCTTCGG

CGATGACCTATGGTAGTTGTCTAACTTCGTATGCGGCTCGATACGTCAATGGTCGGTTGC

ATGCTCAAGGGAAACGACTGCTTCGTTCTTCGTCGTGAGGCAAAATGGAGTTGCTCGCCT

TCAGAGGCTTGTTAGGGCTGGCTTGATTCGTCTTGCACCCTGGCTGTG-CCGGAGGGTGA

GTGGCTTGAGGGGCTTTGTGCGATTTTGCCAAAATGGTTTTTACCGACCCGTCTTGAAAC

ACGGACCAAGGAGTCTAACATGTATGCGAGTATGCTGGTGGAGAAACCAACATGCACAAC

TAACGTGAGCGATGCCAAGCGCAAGCAGCAGCATCGGCCGACCATGATTCTCTGATGAAA

GGATCGCGCAAGAGCATACCTGTTAGGACCCGAAAGATGGTGAACTATGCCTGAGCAGGG

TGAAGCCAGGGGAAACTCTGGTGGAGGCTCGTAGCGACACTGACGTGCAAATCGTTCGTC

TGACTTGGGTATAGGGGCGAAAGACTAATCGAACCATCTAGTAGCTGGTTCCCTCCGAAG

TTTCCCTCAGGATAGCAAGGACAATTATGCAGTTTTATTAGGTAAAGCGAATGATTAGAG

GCCTCGGGGATCCAGCATCCTCGACCTATTCTCAAACTTTAAATTGGTAAGAACCCTGGC

CTTCCTTAATTGAGGCGCAGGGGCTCAATGCGTGTCCTTAGTGGGCCATTTTTGGTAAGC

AGAACTGGCGATGAGGGGTGCTCCTAACGTCGAGTTAAGGTGCCGAAGTGCACGCTCATC

AG-ACACTACAAAGGGTGTTGGTTCATAAAGACAGCAGGACGGTGGCCATGGAAGTCGGA

ATCCGCTAAGGAGTGTGTAACAACTCACCTGCCGAATGAACTAGCCCCGAAAATGGATGG

CGCTTAAGCGTGCGACCGATACTCGACCATTGCGGCGAGAGTGAGGCTGCAATGAGTAGG

AGGGCGTGAGCGTTGTTGTGAAGCCTCTGACGTGAGTCTGGGTTGAACAGCGTTTAGTGC

GGATCTTGGTGGTAGTAGCAATTATTCAAATGAGAACTTTGAAGGCCGAAGTGGAGAAGG

TTTCCGTGAGAACAGCAATTGGTCACGGGTGACTCGATCCTAAGACATGGGGGAAATCCT

TGTTAAGTGCGCCATTTGGT---GCGAAGTCGAAAGGGAATGAGGTTAATATTCCTCAAG

CTGGATGTGGATATTGTATGGCAACATCAATGAGCTCAGAGACGCCAGCGTGAGCCTCTG

GAAGAGTTCTCTTTTCTTTTTAACAGACTAGCGACCTTGAAATTGGATTACCCAGAGCTA

AGGTTGTACGTCTGGAAGAGCACCTCGCTTTTCGAGGTGTCAGGTGCGCTCACGATGGTC

CTTGAAAATCTGAGTGAGAGTT

**>Spirotrachelostyla_tani**

CCAGTAACGGCGAGTGAAGCGGCAAGAGCCCAACATGAAAATCTTCGTGC-TTTAGTACG

ACGAGTTGTACAGTAGAGGGGAGTTCCTCAACCGGCGACGGCGCAGAAGTGCCTTAGAAC

GGGCGGCCATAGAGGGTGACAGCCCCGTATGTGGTGCTATCGCCTAACGAGGGATTTTCC

GAAGAGTCGGGTTGTTTTGTATTGCAGCCCTAAATGGGAGATAAACTTCTTCTAAGGCTA

AATATGTACGGGAAACCGATAGCGAACAAGTACTGTGAAGGAAAGATGAAAAGAACTTTG

AAAAGAGAGTCAAAAGACTTGAAATTGTTGAAAAGGAAGCGGTAGAAATTTATTCTGCGG

AGGTGAACTATCGCCGCTGTCTAACTTCTGGTGGTCGCATGCACTTTAACGGCTGCGTCT

GTCATCAAGGGACACGGCTGCGTTGTTCTTCTCTGTGAGGCAAAATGGAGTTACTTTCTC

GCAAAAGCTTGCTAGGGCTGGCTCGGCTTGCCGGGCACCCTGGCTGTG-CTGGTGAGGAA

GTGGCTTGAGGGGCTACGTGCGATTTTGCCAAAATGGTTTTTACCGACCCGTCTTGAAAC

ACGGACCAAGGAGTCTAACCTGTATGCGAGTATGCTGGTGGAGAAACCAACATGCATAAC

TAACGTGAGCGATGCCAAGCGCAAGCAGCAGCATCGGCCGACCATGATTCTCTGATGAAA

GGTTCGAGCAAGAGCATACCTGTTAGGACCCGAAAGATGGTGAACTATGCCTGAGCAGGG

TGAAGCCAGGGGAAACTCTGGTGGAAGCTCGAAGCGATACTGACGTGCAAATCGTTCGTC

TGACTTGGGTATAGGGGCGAAAGACTAATCGAACCATCTAGTAGCTGGTTCCCTCCGAAG

TTTCCCTCAGGATAGCAAGGACAATAATGCAGTTTTATTAGGTAAAGCGAATGATTAGAG

GCCTCGGGGGCCCAGTGCCCTCGACCTATTCTCAAACTTTAAATTGGTAAGAACCCTGGC

CTTCCTTAATTGAGGCGCAGGGGCTCAATGCGTGTCCTTAGTGGGCCATTTTTGGTAAGC

AGAACTGGCGATGAGGGATGCTCCTAACGTCGAGTTAAGGTGCCTAAGTGCACGCTCATC

AGCACACTGAAAAGGGCGTTGGTTCATACAGACAGCAGGACGGTGGCCATGGAAGTCGGA

ACCCGCTAAGGAGTGTGTAACAACTCACCTGCCGAATGAACCAGCCCCGAAAATTGATGG

CGCTTAAGCGTACGACCGATACTTGACCATTGCAGCGAGAGTGAGGCCGCAATGAGTAGG

AGGGCGTGAGCGTTGTTGTGAAGCCTCTGGCGTGAGCCTGGGTTGAACAGCGTTTAGTGC

AGATCTTGGTGGTAGTAGCAATTATTCAAATGAGAACTTTGAAGGCCGAAGTGGAGAAGG

TTTCCGTGAGAACAGCAATTGGTCACGGGTGACTCGATCCTAAGACATGGGGGAAATCCT

TGTTAAGTGCCGTGATTGCATCGGCGAAGTCGAAAGGGAAAGAGGTTAATATTCCTCTAG

CTGGATGTGGATTTTGTATGGCAACATAAATGAGCTCAGAGACGCCAGCGTGAGCCTCTG

GAAGAGTTCTCTTTTCTTTTTAACAGACTAGCGACCTTGAAATTGGATTACCCAGAGCTA

AGGTTGTACGTTTGGAAGAGCACCTCGCTTTTCGAGGTGTCAGGTGCGCTCACGATGGCC

CTTGAAAATCTGAGTGAGAGTT
